# IL-33, diet-induced obesity, and pulmonary responses to ozone

**DOI:** 10.1186/s12931-020-01361-9

**Published:** 2020-04-23

**Authors:** David I. Kasahara, Stephanie A. Shore

**Affiliations:** Department of Environmental Health, Molecular and Integrative Physiological Sciences Program, Harvard T.H. Chan School of Public Health, 665 Huntington Avenue, Boston, MA 02115-6021 USA

**Keywords:** High fat diet, Airway responsiveness, Neutrophil, IL-5, Microbiome

## Abstract

**Background:**

Obesity augments pulmonary responses to ozone. We have reported that IL-33 contributes to these effects of obesity in *db/db* mice. The purpose of this study was to determine whether IL-33 also contributes to obesity-related changes in the response to ozone in mice with diet-induced obesity.

**Methods:**

Male wildtype C57BL/6 mice and mice deficient in ST2, the IL-33 receptor, were placed on chow or high fat diets for 12 weeks from weaning. Because the microbiome has been implicated in obesity-related changes in the pulmonary response to ozone, mice were either housed with other mice of the same genotype (same housed) or with mice of the opposite genotype (cohoused). Cohousing transfers the gut microbiome from one mouse to its cagemates.

**Results:**

Diet-induced increases in body mass were not affected by ST2 deficiency or cohousing. In same housed mice, ST2 deficiency reduced ozone-induced airway hyperresponsiveness and neutrophil recruitment in chow-fed but not HFD-fed mice even though ST2 deficiency reduced bronchoalveolar lavage IL-5 in both diet groups. In chow-fed mice, cohousing abolished ST2-related reductions in ozone-induced airway hyperresponsiveness and neutrophil recruitment, but in HFD-fed mice, no effect of cohousing on these responses to ozone was observed. In chow-fed mice, ST2 deficiency and cohousing caused changes in the gut microbiome. High fat diet-feeding caused marked changes in the gut microbiome and overrode both ST2-related and cohousing-related differences in the gut microbiome observed in chow-fed mice.

**Conclusion:**

Our data indicate a role for IL-33 in pulmonary responses to ozone in chow-fed but not high fat diet-fed mice and are consistent with the hypothesis that these diet-related differences in the role of IL-33 are the result of changes in the gut microbiome.

## Introduction

Obesity is increasingly recognized as an important risk factor for asthma. Obesity increases both the prevalence and incidence of asthma [1]. Obesity also increases the severity of asthma and reduces the efficacy of standard asthma control medications [2, 3]. Furthermore, in obese asthmatics, weight loss improves asthma symptoms and reduces airway hyperresponsiveness (AHR), a canonical feature of asthma [4, 5]. These effects of obesity are particularly prominent in non-atopic asthmatics [5].

Ozone (O_3_), a common air pollutant generated from automobile exhaust in the presence of sunlight, is a non-atopic asthma trigger. Exposure to O_3_ reduces lung function, causes AHR, and induces symptoms of asthma [6–8]. Hospital admissions and emergency room visits for asthma are higher after days when environmental O_3_ concentrations are elevated [6, 7]. Both overweight and obesity exacerbate O_3_-induced decrements in lung function [9, 10] and the effects of obesity on O_3_-induced changes in lung function are magnified in subjects with pre-existing AHR [9]. Pulmonary effects of acute O_3_ exposure, including airway obstruction and AHR, are also greater in obese than lean mice [11, 12]. These observations suggest a link between obesity, responses to air pollution, and asthma. Greater understanding of this link could lead to improved therapeutic options for the obese asthmatic population.

We have reported that IL-33, a member of the IL-1 cytokine family, contributes to the pulmonary responses to acute O_3_ exposure in obese *db/db* mice [12]. IL-33 and its receptor, ST2, are both also genetically linked to asthma [13]. IL-33 is expressed in airway epithelial cells and is released upon cell necrosis [14], including after O_3_-induced injury [15]. Indeed, bronchoalveolar lavage (BAL) fluid concentrations of IL-33 increase following acute O_3_ exposure and these increases are greater in *db/db* than lean mice [12]. Furthermore, in *db/db* mice, treatment with a blocking antibody to ST2 attenuates O_3_-induced airway obstruction, O_3_-induced AHR, and also attenuates O_3_-induced neutrophil recruitment to the lungs [12].

While the study described above indicates an important role for IL-33 in the ability of obesity to augment responses to O_3_, the study was limited to obese *db/db* mice. These mice are obese because of a genetic deficiency in the receptor for leptin, a satiety hormone. The purpose of the study described herein was to examine the hypothesis that IL-33 also contributes to the effects of O_3_ in mice with diet-induced obesity (DIO) caused by high fat diet (HFD) feeding. Mice with DIO have intact leptin receptors and increased circulating leptin [16], a situation similar to that observed in human obesity. Therefore, we placed weanling wildtype (WT) mice and mice with a genetic deficiency in ST2 (ST2^−/−^ mice) either on regular chow diets or on diets in which 60% of the calories derived from fat in the form of lard. Diets were maintained for 12–14 weeks. Mice were then exposed to O_3_ (2 ppm) or room air for 3 h. Our results indicate reductions in O_3_-induced AHR and neutrophil recruitment in ST2 deficient versus WT chow-fed but not HFD-fed mice.

We have recently reported a role for the microbiome in the effects of ST2 deficiency on O_3_-induced AHR in lean male mice: compared to WT mice, ST2 deficient mice housed with other ST2-deficient mice (same housed mice) have reduced O_3_-induced AHR, but housing ST2 deficient mice with WT mice (cohousing) reverts the magnitude of their O_3_-induced to that observed in WT mice [17]. Because mice ingest some of the fecal microbiota of their cagemates either during grooming or as a result of coprophagy, cohousing transfers gut microbiota from one mouse to its cagemates and rapidly normalizes differences in their gut microbiota [18, 19]. Indeed, we observed effects of both ST2 deficiency and effects of cohousing on the gut microbial community structures of these lean chow-fed mice [17]. Consistent with these observations, others have reported differences in the gut microbiomes of IL-33 deficient and WT mice [18]. The data suggest a role for the microbiome in mediating effects of ST2 deficiency on pulmonary responses to O_3_ in lean male mice. HFD feeding causes marked changes in the gut microbiome [20, 21]. Moreover, the gut microbiome contributes to obesity-related increases in pulmonary responses to O_3_ [22]. To determine whether the microbiome might also contribute to differences in the impact of ST2 deficiency on pulmonary responses to O_3_ in chow-fed versus HFD-fed mice, effects of ST2 deficiency on O_3_-induced AHR and inflammation were examined in both same housed and cohoused mice fed HFD. Our data indicate that whereas cohousing attenuates effects of ST2 deficiency on O_3_- induced AHR and neutrophil recruitment in chow-fed mice, cohousing has no effect on responses to O_3_ in HFD-fed mice, likely because HFD-feeding overrides ST2- and cohousing-related changes in the gut microbiome.

## Methods

### Animals

**Fig. 1:**
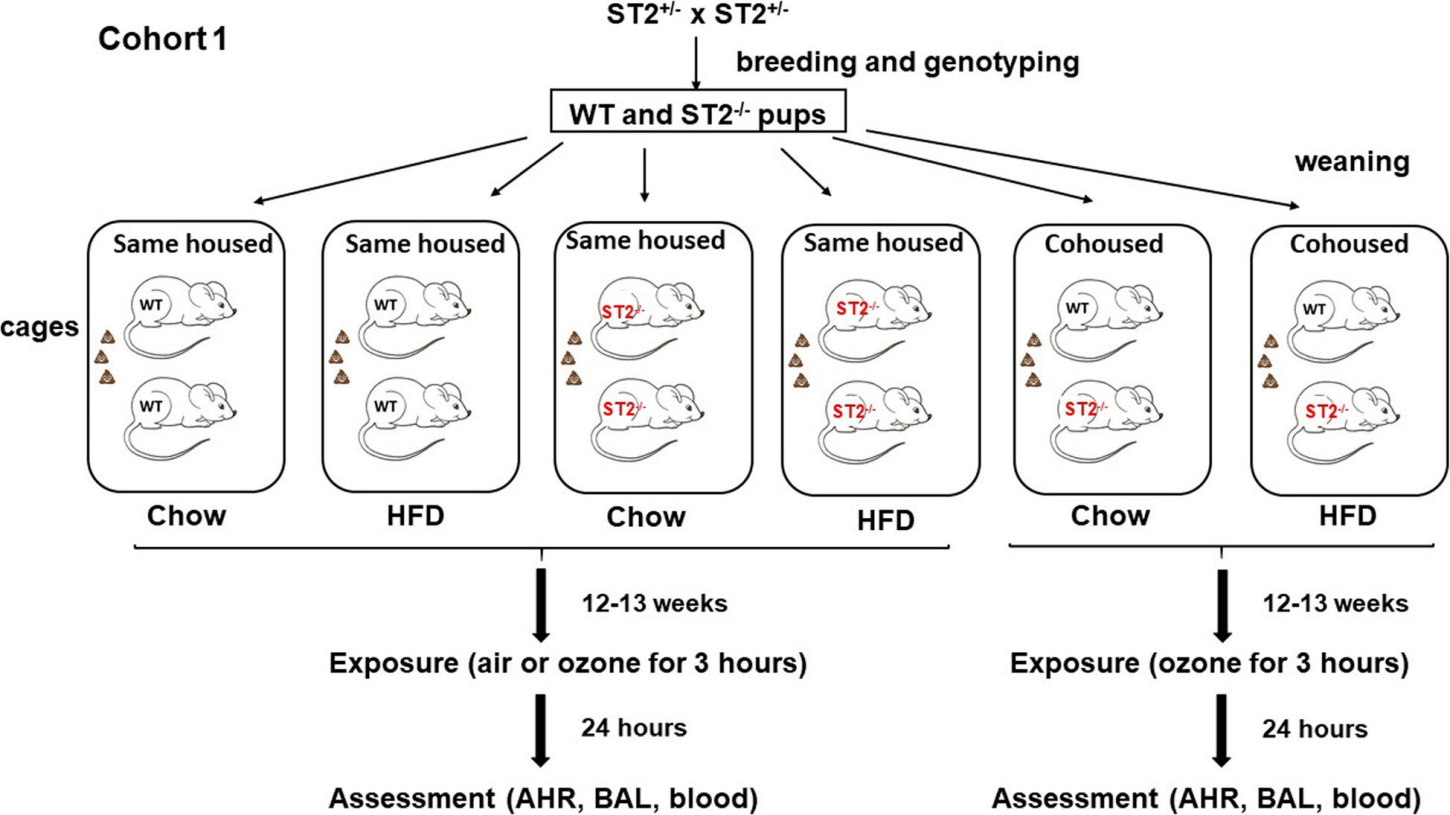
Schematic representation of study protocol for cohort 1. Male WT and ST2^−/−^ pups from ST2^+/−^ parents were placed at weaning in cages with other mice of the same genotype (same housed) or with mice of the opposite genotype (cohoused). Cages from each genotype/housing combination were assigned to either chow or high fat diet (HFD). After 12–13 weeks on the diet, mice were exposed to ozone (2 ppm) or air for 3 h. 24 h later, mice were anesthetized for the measurement of airway hyperresponsiveness (AHR). A bronchoalveolar lavage (BAL) was performed and blood was collected

This study was approved by the Harvard Medical Area Standing Committee on Animals. ST2^−/−^ mice on a C57BL/6 background were generated by Dr. Andrew McKenzie at Cambridge University [23] and obtained, with permission, from a colony at Yale School of Medicine. These ST2^−/−^ mice were bred with C57BL/6 J mice purchased from The Jackson Labs (Bar Harbor, ME) to obtain ST2^+/−^ mice. ST2^+/−^ mice were bred together to obtain most of the ST2^−/−^ and WT mice used for these experiments. Some additional WT and ST2^−/−^ offspring of the ST2^+/−^ parents were used as breeders to generate other WT and ST2^−/−^ mice used in these experiments. Mice were weaned at 3–4 weeks of age, and the male mice were assigned to one of two caging strategies as outlined in Fig. 1. Mice were either housed with other mice of the same genotype (same housed) or were cohoused with mice of the opposite genotype (cohoused), as previously described [17]. Male mice were used because female mice are resistant to the induction of obesity by HFD feeding [24]. Cage changes for all mice were performed weekly by the same investigator. Data from the chow-fed mice were previously described [17] and are included here because these mice were the siblings of the mice studied after HFD feeding and hence are the appropriate controls for the HFD-fed mice. All mice were exposed to a 12 h light:dark cycle (6 AM,6 PM).

### Protocol

After weaning, at approximately 4 weeks of age, male WT and ST2^−/−^ mice were placed on either a high fat diet (HFD) in which 60% of calories derived from fat in the form of lard (D12451, Research Diets Inc.) or on normal mouse chow (PicoLab 5053, LabDiets) in which about 13% of calories derive from fat (see Fig. 1). Mice were maintained on these diets for 12–13 weeks, at which time they were exposed to air or O_3_ (2 ppm for 3 h). In some mice, a fecal pellet was obtained just before exposure and frozen in liquid nitrogen until DNA extraction. Twenty four hours after cessation of exposure, mice were anesthetized for the measurement of pulmonary mechanics and airway responsiveness. After these measurements, mice were euthanized, blood was obtained by cardiac puncture for the preparation of serum and BAL was performed. Serum was prepared from blood and analyzed by ELISA for systemic inflammation using a multiplex assay that measured a variety of cytokines and chemokines (Eve Technologies, Calgary, Alberta).

**Fig. 2:**
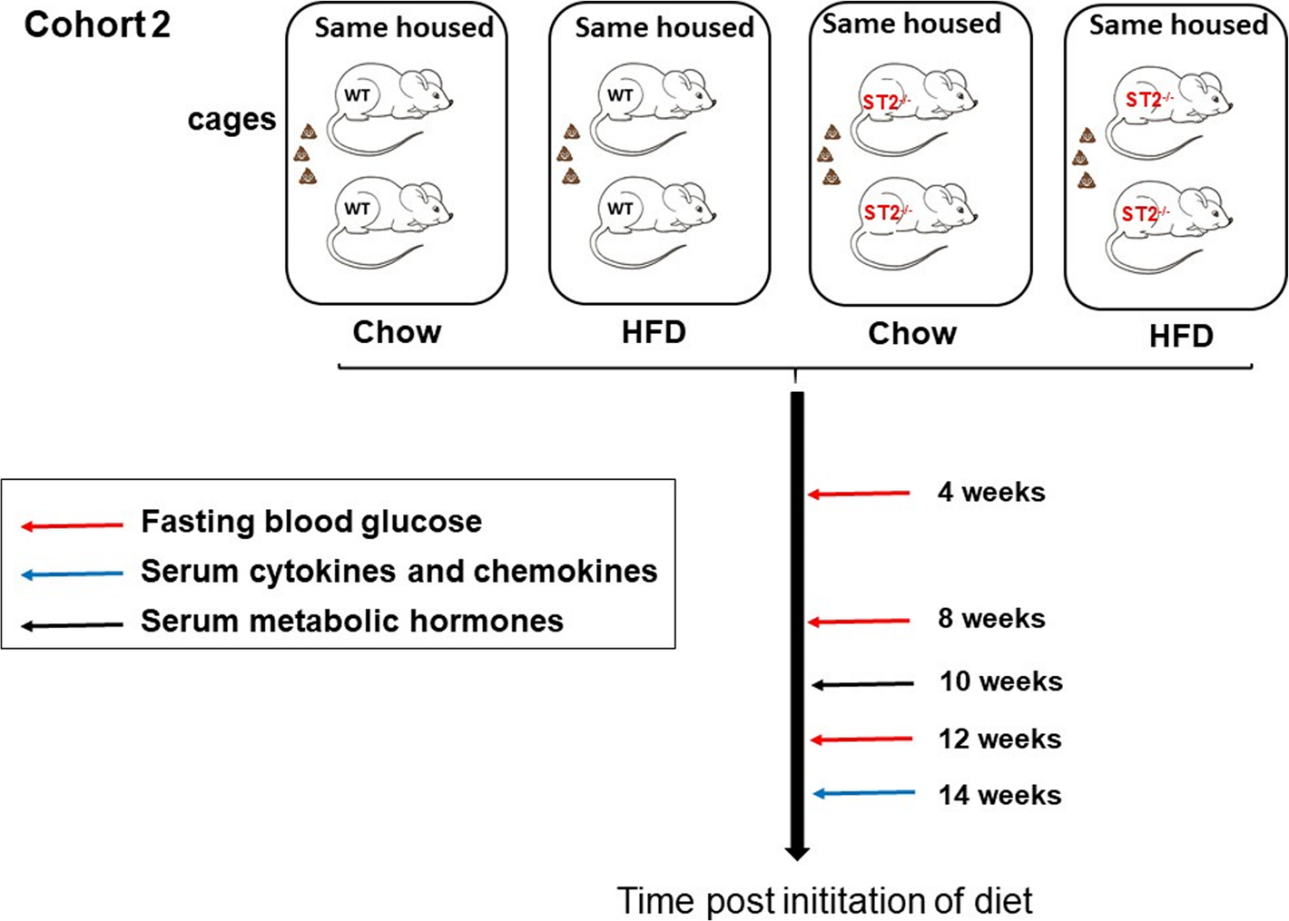
Schematic representation of study protocol for cohort 2. At weaning, male WT and ST2^−/−^ pups were placed in cages with other mice of the same genotype. Each cage was assigned to either chow or HFD. Prior to (not shown) and after 4, 8, and 12 weeks on the diet mice were fasted overnight. A tail nick was then performed for the assessment of fasting blood glucose. After 10 weeks and 14 weeks on the diet, a mandibular blood draw was performed for the assessment of metabolic hormones and serum cytokines and chemokines, respectively

In another cohort of mice (see schematic representation in Fig. 2), we performed an overnight fast at monthly intervals during the induction of obesity. The next morning, a drop of blood was harvested by a small tail nick to evaluate fasting blood glucose (AlphaTRAK, Zoetis, USA). At 14 weeks after initiation of diets, a mandibular blood draw (50–100 ul) was performed to generate serum used to evaluate systemic inflammation using a multiplex assay (Eve Technologies). A mandibular blood draw was also performed at 10 weeks to obtain serum used to evaluate energy-related hormones (Eve Technologies). In this latter cohort, only same housed mice were used.

### Ozone exposure

During exposure to O_3_ (2 ppm for 3 h)*,* mice were placed in individual wire mesh cages within a stainless steel and plexiglass exposure chamber, as described [11] and had no access to food or water. Immediately after exposure, mice were returned to regular cages with free access to food and water. Mice exposed to air were treated identically, but exposed to room air only. During the period between cessation of exposure and anesthesia for the measurement of airway responsiveness (24 h), mice were given the same diets that they had received prior to exposure.

### Measurement of airway responsiveness

Mice were anesthetized with sodium pentobarbital and xylazine for the measurement of pulmonary mechanics and airway responsiveness to inhaled aerosolized methacholine. The trachea was cannulated using a tubing adapter and the mice were ventilated (Flexivent, Scireq) as previously described [17]. The chest wall was opened to expose the lungs to atmospheric pressure and a positive end expiratory pressure of 3 cm H_2_O was imposed. Pulmonary mechanics were assessed using the forced oscillation technique. Changes in total lung resistance (R_L_), Newtonian resistance (Rn), and the coefficients of lung tissue damping (G) and elastance (H) were assessed after aerosolized saline and after each of several doses of aerosolized methacholine increasing in half log increments, as previously described [25].

### Bronchoalveolar lavage

The lungs were lavaged twice with 1 ml of cold PBS and the lavageates pooled. BAL fluid was centrifuged and total BAL cells were counted in a hemacytometer. Cytospin slides were prepared and stained with Hemacolor (EMD-Millipore) to obtain differential cell counts. BAL supernatants were stored at -80 °C until assayed for IL-17A and IL-33 by commercial enzyme linked immunosorbent assay (ELISA) (Biolegend and eBioscience respectively). A multiplex assay (Eve Technologies, Calgary, Alberta) was used to measure other BAL cytokines and chemokines. For the multiplex assay, BAL supernatants were concentrated approximately 8-fold using Amicon Ultra-0.5 centrifugal filters (Ultra 3kda, EMD-Millipore) prior to analysis. Concentrations reported are those present in the original samples. BAL protein, a marker of O_3_-induced lung injury, was assayed using the Bicinchoninic acid method (Pierce-Thermo Fischer, Rockford, IL).

### Fecal DNA extraction

Immediately before mice were exposed to air or ozone, fecal pellets were collected and stored at -20 °C. For chow-fed mice, total DNA was extracted from fecal pellets as previously described [17]. For HFD-fed mice, fecal DNA was extracted using the Quick DNA fecal/soil microbe microprep kit (Zymo Research, CA, USA). Fecal DNA concentration and quality were measured with a Nanodrop (ThermoFisher, Waltham, MA).

### 16S rRNA sequencing and analysis

16S rRNA gene sequencing of fecal DNA was performed to evaluate differences in the gut microbial community structures induced by ST2 deficiency, cohousing, and HFD feeding. Sequencing was performed at the Massachusetts Host-Microbiome Center at Brigham and Women’s Hospital as previously described [17]. The resulting sequencing data (FastaQ) data were analyzed as follows. Forward and reverse sequences as well as the metadata file were submitted to NIH/NIAID Nephele microbiome analysis platform (https://nephele.niaid.nih.gov) using the Qiime pipeline for all taxonomic outputs. Sequencing raw data (Fastaq) and metadata files have been deposited at the National Institute of Health – Sequence Read Archive (SRA) with accession number PRJNA516522 (biosample_accession SAMN10790706 – SAMN10790770). We used Multivariate Association with Linear Model - MaAsLin [26] (http://huttenhower.org/galaxy) to assess the statistical significance of differences in taxon abundance among both housing, genotype, and diet factors in arcsine-square root transformed relative abundance data. Data with *p* < 0.05 and q < 0.25 were considered statistically significant.

### Statistics

Except where indicated, data were analyzed by factorial ANOVA using STATISTICA software (StatSoft®; Tulsa, OK) with mouse genotype, housing, exposure, and diet as main effects. Fisher’s LSD test was used as a post-hoc test. BAL cells were log transformed prior to analysis in order to conform to a normal distribution. A *p* value < 0.05 was considered statistically significant.

## Results

### Effects of ST2 deficiency on HFD-induced changes in body mass, insulin resistance, and systemic inflammation

**Fig. 3:**
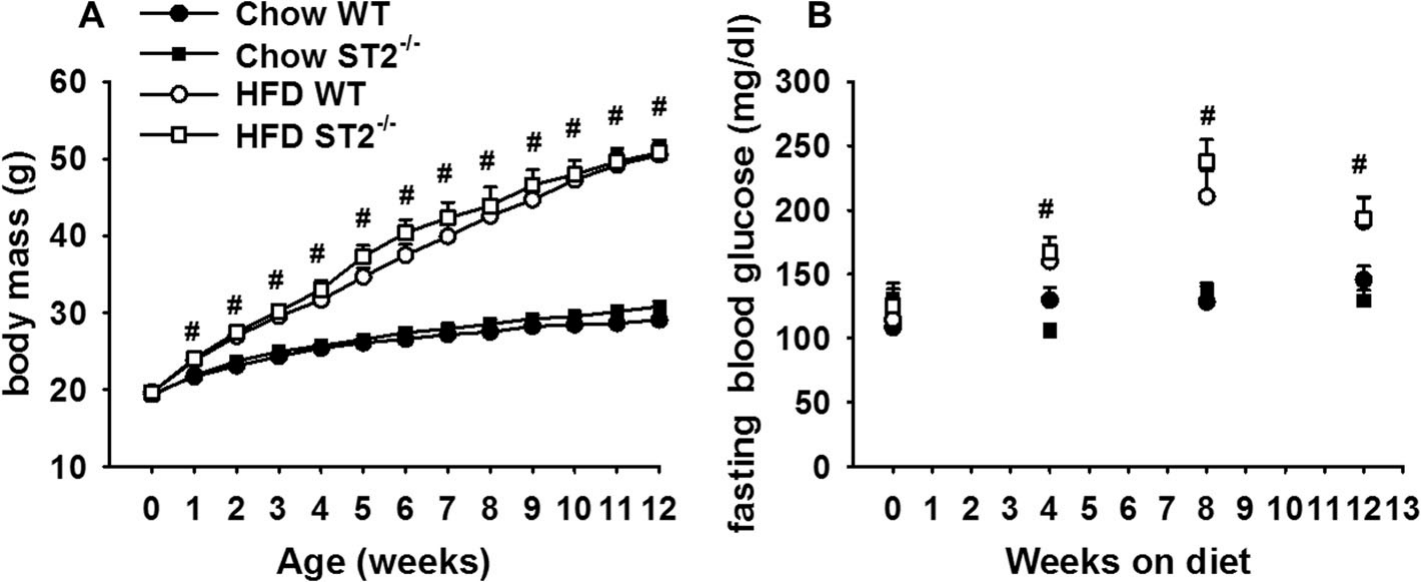
Effect of HFD and ST2 deficiency on (**a**) body mass and (**b**) fasting blood glucose. At weaning, wildtype (WT) and ST2 deficient (ST2^−/−^) mice were placed either on high fat diets (HFD) in which 60% of calories derived from fat in the form of lard, or on regular chow. Mice were housed with other mice of the same genotype. Results are mean + SE of 6–19 mice/group. # Factorial ANOVA indicated a significant (*p* < 0.05) effect of diet

In C57BL/6 male mice, HFD feeding causes a metabolic syndrome that includes increases in body mass, insulin resistance, and systemic inflammation [27]. Indeed, compared to chow-fed mice, HFD-fed mice gained substantially more weight over the 12 week feeding protocol (Fig. 3a). This weight gain occurred rapidly: even 1 week after diet initiation there was a significant difference in body mass in the HFD- versus chow-fed mice and by 12 weeks on the diets, the HFD-fed mice weighed approximately 60% more than the chow-fed mice. There was no effect of ST2 deficiency on the magnitude or time course of HFD-induced weight gain.

**Fig. 4:**
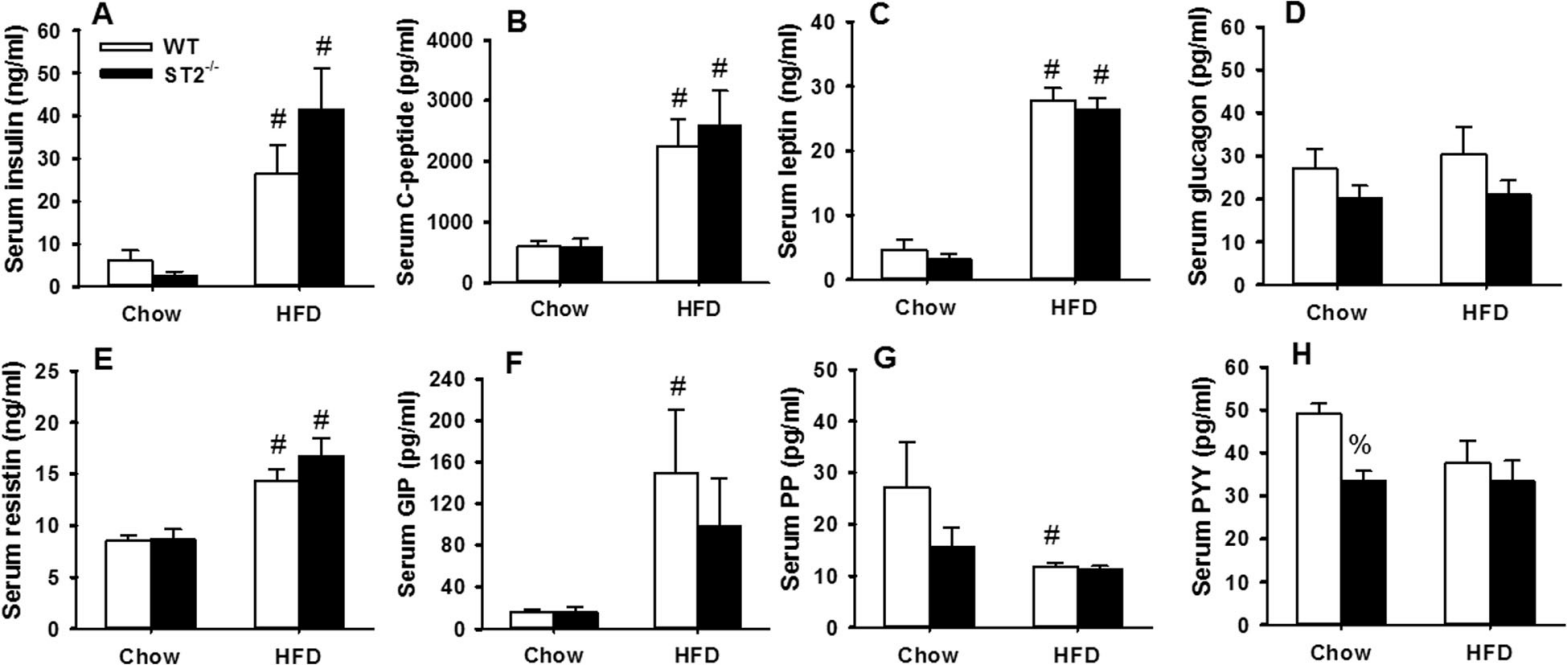
Effect of HFD and ST2 deficiency on eating- and energy-related hormones. Shown are serum concentrations of **a** insulin, **b** C-peptide, **c** leptin, **d** glucagon, **e** resistin, **f** gastric inhibitory polypeptide (GIP), **g** pancreatic polypeptide (PP), and **h**) peptide YY (PYY) in unexposed WT and ST2^−/−^ mice that were fed chow or HFD for 10 weeks from weaning. Mice were housed with other mice of the same genotype. Results are the mean + SE of 5–7 mice/group. # *p* < 0.05 versus chow-fed mice with same genotype; % *p* < 0.05 versus WT mice with the same diet

**Fig. 5:**
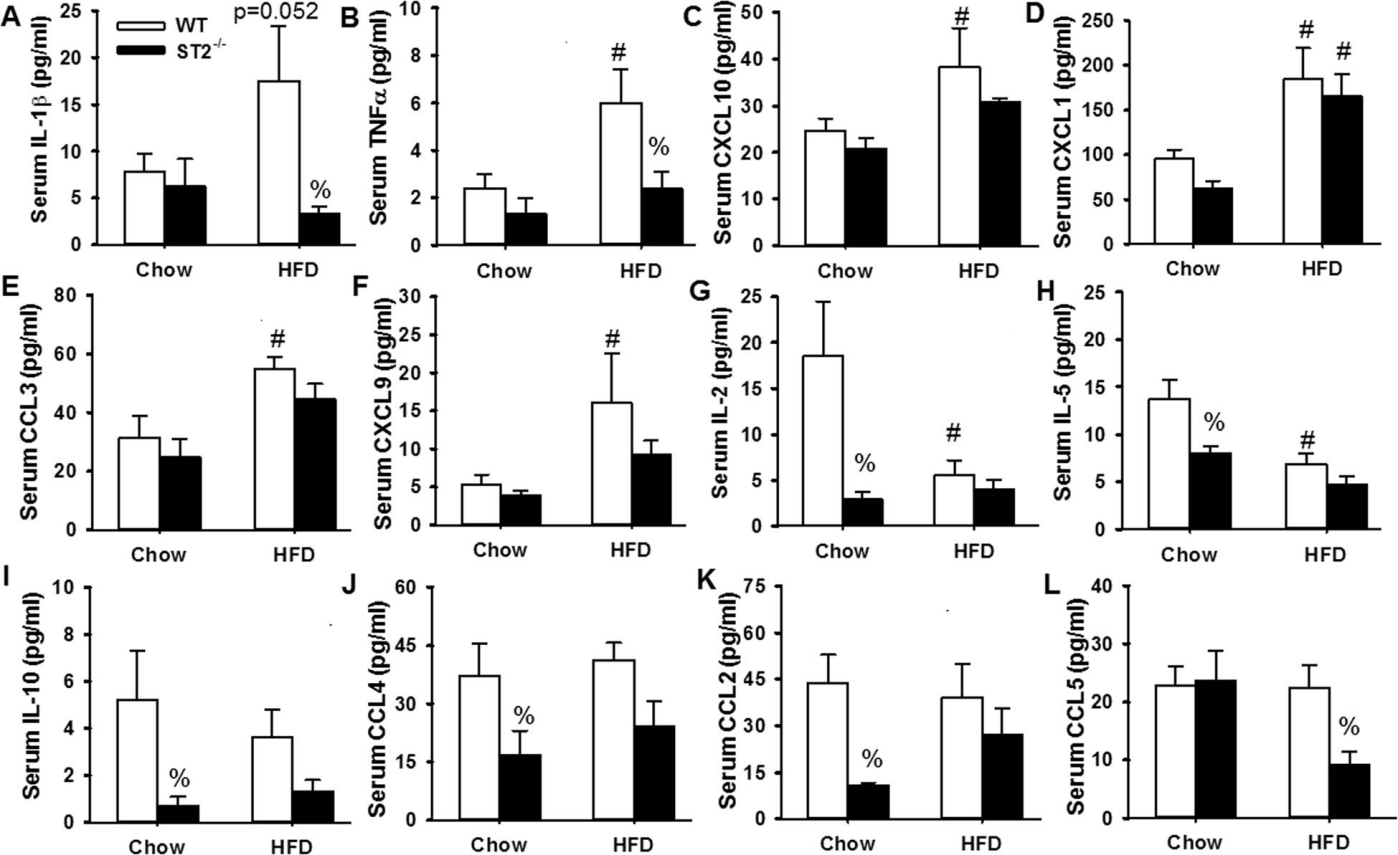
Effect of HFD and ST2 deficiency on markers of systemic inflammation. Shown are serum concentrations of **a** IL-1β, **b** TNFα, **c** CXCL10, **d** CXCL1, **e** CCL3, **f** CXCL9, **g** IL-2, **h** IL-5, **i** IL-10, **j** CCL4, **k** CCL2, and **l** CCL5 in unexposed WT and ST2^−/−^ mice that were fed chow or HFD for 14 weeks from weaning. Mice were housed with other mice of the same genotype. Results are the mean + SE of 5–7 mice/group. # *p* < 0.05 versus chow-fed mice with same genotype; % *p* < 0.05 versus WT mice with the same diet

In a separate cohort of unexposed mice, fasting blood glucose was assessed prior to and 4, 8, and 12 weeks after the onset of HFD feeding (Fig. 3b). In the same cohort, a mandibular blood draw was performed after 10 or 14 weeks of feeding for measurement of serum concentrations of hormones known to affect eating and metabolism (Fig. 4) and to evaluate systemic inflammation (Fig. 5) using multiplex assays. Compared to chow-fed mice, fasting blood glucose was greater in HFD-fed mice at all time points after the onset of HFD feeding (Fig. 3b). There was no effect of ST2 deficiency on the magnitude or time course of HFD-induced increases in fasting glucose. Compared to chow, HFD feeding caused significant increases in serum concentrations of insulin, C-peptide, leptin, resistin, and gastrin inhibitory peptide (GIP), while pancreatic polypeptide (PP) was reduced (Fig. 4). Compared to WT mice, chow fed ST2^−/−^ mice had significantly lower concentrations of peptide YY (PYY), but no other hormones were significantly affected by ST2 deficiency in chow-fed mice. There were no effects of ST2 deficiency on serum hormones in HFD-fed mice although greater increases in serum insulin in ST2^−/−^ than WT mice (Fig. 4a) were near significance (0.05 < *p* < 0.10).

Obesity causes a state of low grade systemic inflammation that contributes to some of the physiological effects of this condition [28]. In order to assess the impact of ST2 deficiency on the systemic inflammation of obesity, a multiplex assay was used to assess a panel of cytokines and chemokines in serum. Serum cytokines and chemokines that were significantly altered by either HFD or ST2 deficiency are shown in Fig. 5. Compared to chow-fed WT mice, HFD-fed WT mice had increases in serum concentrations of the pro-inflammatory cytokines and chemokines IL-1β, TNFα, CXCL10, CXCL1, CCL3, and CXCL9 (Fig. 5a-f). In contrast, serum concentrations of IL-2 and IL-5 were lower in WT HFD-fed than WT chow-fed mice (Fig. 5g, h). Importantly, in chow-fed mice, ST2 deficiency caused significant reductions in serum IL-2, IL-5, IL-10, CCL4, and CCL2 (Fig. 5g-k). ST2 deficiency did not significantly alter any of these cytokines in HFD-fed mice. However, ST2 deficiency did cause significant reductions in serum IL-1β, TNFα, and CCL5 in the HFD-fed mice (Fig. 5a, b, l), suggesting that IL-33 contributes to aspects of the systemic inflammation of obesity.

### Effects of ST2 deficiency on pulmonary responses to O_3_ in same housed mice

In the description immediately below, we refer only to same-housed mice. Results obtained in cohoused mice are found in the section below this one.

#### Pulmonary mechanics and airway responsiveness

##### Air exposed mice

**Fig. 6:**
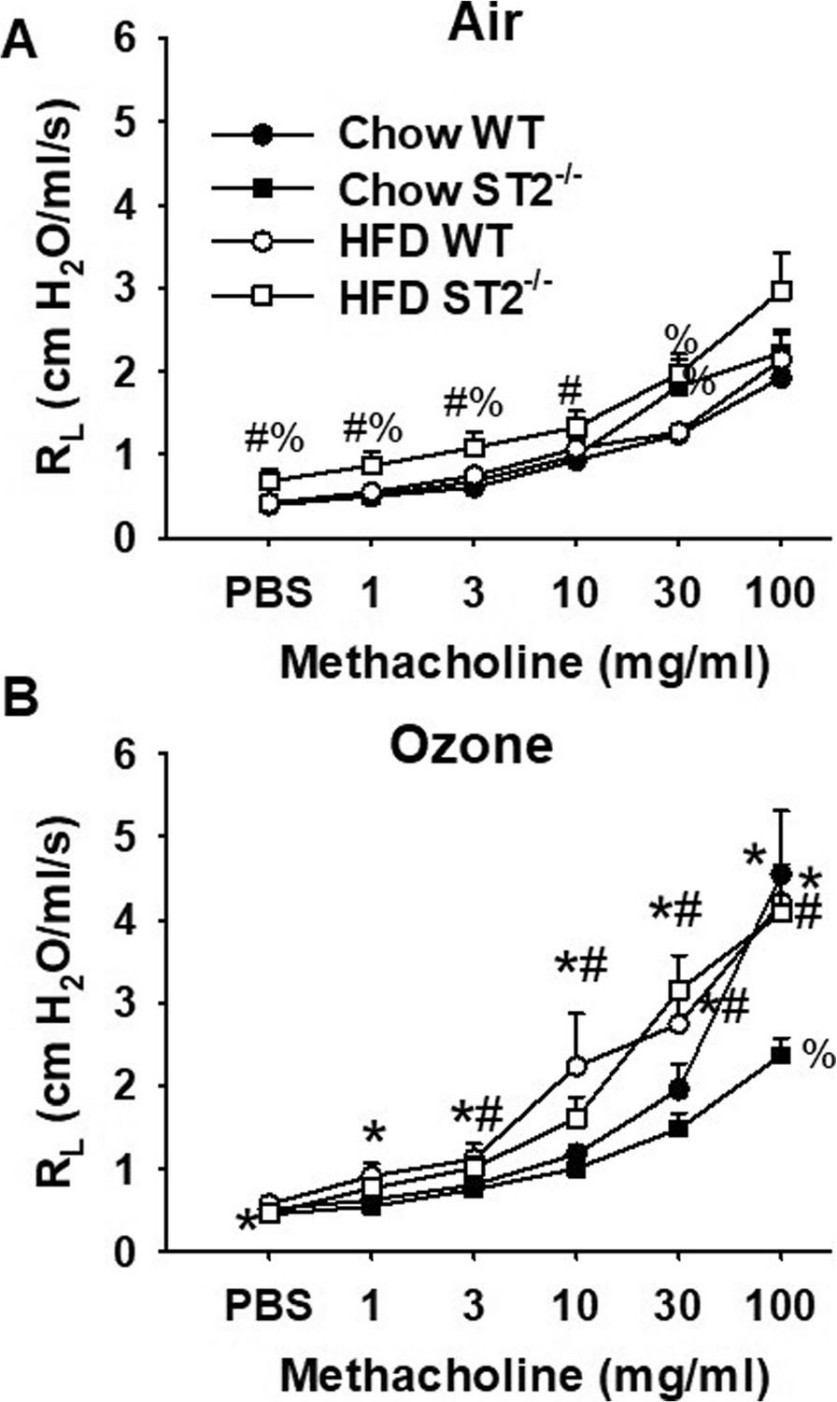
Effect of HFD and ST2 deficiency on O_3_-induced airway hyperresponsiveness. Shown are changes in pulmonary resistance (R_L_) induced by inhaled aerosolized methacholine in WT and ST2^−/−^ mice exposed to (**a**) air or (**b**) O_3_ (2 ppm for 3 h) and examined 24 h after exposure. All mice were same housed, as outlined in Fig. 1. Results are mean + SE of 6–11 mice/group. * *p* < 0.05 versus air exposed mice of same genotype and diet; # *p* < 0.05 versus chow fed mice with same genotype and exposure; % *p* < 0.05 versus WT mice with same exposure and diet

In chow-fed mice, there was no effect of ST2 deficiency on airway responsiveness, as described [17] (Fig. 6a). In WT mice, 12–13 weeks of HFD feeding had no effect on airway responsiveness. However, compared to HFD-fed WT mice, airway responsiveness was *increased* in HFD-fed ST2^−/−^ mice, primarily as a result of increased baseline R_L_ (PBS dose in Fig. 6a, Fig. S1). These changes in airway responsiveness were primarily the results of effects within the central airways rather than the lung periphery, since Rn but not G or H was also affected by ST2 deficiency in the HFD-fed mice (Fig. S2A-C). The data are consistent with the hypothesis that IL-33 protects against the development of innate AHR in mice with diet-induced obesity.

##### O_3_-exposed mice

O_3_ had no significant effect on baseline R_L_ in either WT or ST2^−/−^ same housed chow-fed mice (Fig. S1). In contrast, in same-housed HFD-fed mice, O_3_ significantly increased baseline R_L_ in WT mice, but significantly reduced R_L_ in ST2^−/−^ mice (Fig. S1). O_3_-induced increases in R_L_ in the HFD-fed WT mice were primarily the result of effects of O_3_ in the lung periphery rather than the central airways, since G and H but not Rn were also increased by O_3_ exposure (Fig. S1).

Compared to air, airway responsiveness was increased 24 h after exposure to O_3_ in chow-fed WT mice (Fig. 6b). Compared to chow-fed WT mice, chow-fed ST2^−/−^ mice had reduced airway responsiveness (Fig. 6b), as we have described [17]. Indeed, O_3_ exposure failed to increase airway responsiveness in the chow-fed ST2^−/−^ mice. Compared to chow-fed WT mice, O_3_-exposed HFD-fed WT mice had greater airway responsiveness as indicated by significantly greater R_L_ values throughout the middle part of the dose response curve (Fig. 6b). Similar results were obtained in HFD-fed ST2^−/−^ mice and in contrast to the reduced airway responsiveness observed in chow-fed ST2^−/−^ versus WT mice, there was no effect of ST2 deficiency on airway responsiveness in HFD-fed mice (Fig. 6b). The more pronounced O_3_-induced AHR observed in the HFD- than the chow fed mice (Fig. 6b) was primarily the result of differences in G and H, suggesting effects in the peripheral lung, though there were also smaller effects of HFD on Rn (Fig. S2). Thus, HFD feeding abolished the beneficial effects of ST2 deficiency on O_3_-induced AHR observed in chow-fed mice.

##### Pulmonary injury and inflammation

**Fig. 7:**
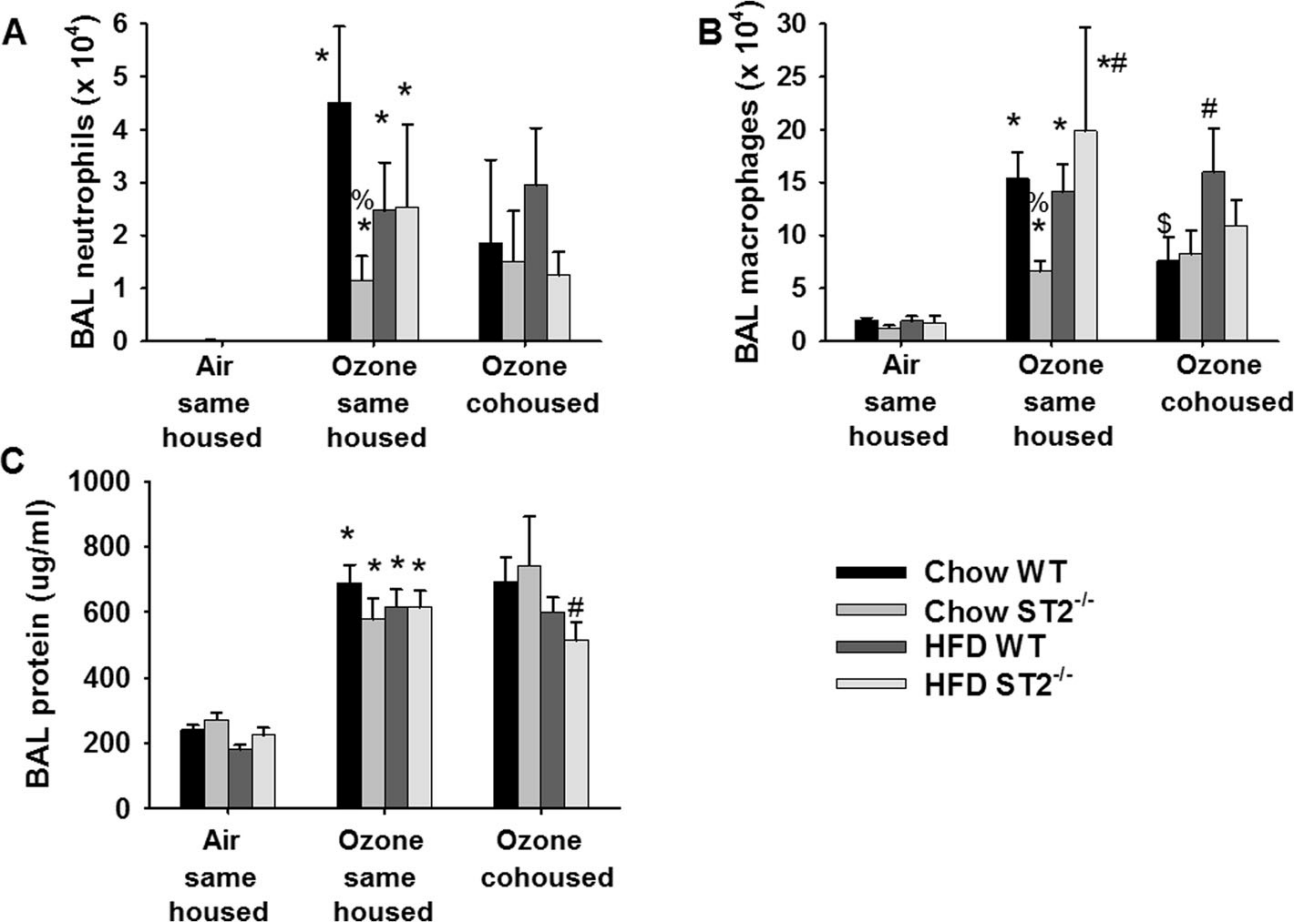
Effect of HFD, ST2 deficiency, and cohousing on O_3_-induced increases in BAL cells and protein. Shown are **a** bronchoalveolar lavage (BAL) neutrophils, **b** macrophages, and **c** protein, a marker of injury to the alveolar/capillary barrier, in WT and ST2^−/−^ fed chow or HFD for 12 weeks after weaning and exposed to air or O_3_ (2 ppm for h). For the O_3_-exposed mice, mice were housed with other mice of the same or opposite (cohoused) genotype. Results are mean + SE of 6–11 mice/group. * *p* < 0.05 versus air exposed mice of same genotype, diet, and housing; # *p* < 0.05 versus chow fed mice with same exposure, genotype, and housing; % *p* < 0.05 versus WT mice with same exposure and housing and diet; $ *p* < 0.04 versus same housed mice with the same exposure, genotype, and diet. Note that only same housed mice were studied with air exposure

Compared to air, O_3_ caused a significant increase in the numbers of neutrophils and macrophages in BAL fluid as well as increased concentrations of BAL protein, a marker of injury to the alveolar/capillary barrier (Fig. 7a-c). In chow-fed mice, ST2 deficiency caused a reduction in BAL neutrophils and macrophages, as we have reported [17]. In contrast, ST2 deficiency had no effect on BAL neutrophils or macrophages in HFD-fed mice (Fig. 7a, b). There was no significant effect of HFD- versus chow-feeding on BAL neutrophils or macrophages in WT mice, but BAL macrophages were significantly greater in HFD- versus chow-fed ST2^−/−^ mice. Neither HFD feeding nor ST2 deficiency had any effect on BAL protein (Fig. 7c).

**Fig. 8:**
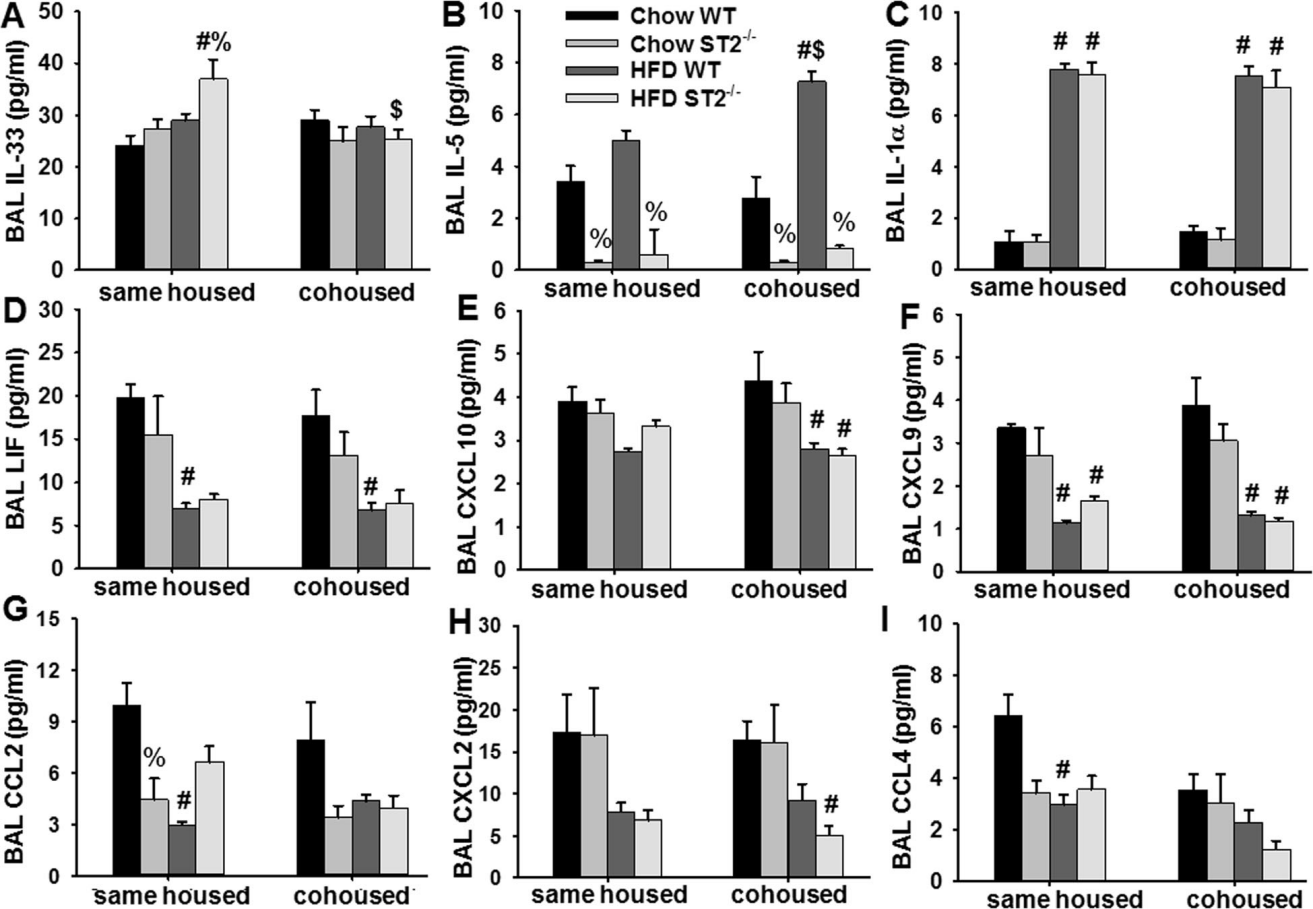
Effect of HFD, ST2 deficiency, and cohousing on BAL inflammatory mediators in O_3_ exposed mice. Shown are BAL concentrations of **a** IL-33, **b** IL-5, **c** IL-1α, **d** LIF, **e** CXCL10, **f** CXCL9, **g** CCL2, **h** CXCL2, and **i** CCL4 in WT and ST2^−/−^ mice fed chow or HFD for 12 weeks from weaning and then exposed to O_3._ Mice were housed with other mice of the same or opposite (cohoused) genotype. Results are the mean + SE of 4–7 mice/group. # *p* < 0.05 versus chow fed mice with same genotype and housing; % *p* < 0.05 versus WT mice with same diet and housing; $ *p* < 0.05 versus same housed mice with the same diet and genotype

O_3_ exposure causes the release of multiple cytokines and chemokines in the lungs, many of which have been implicated in effects of O_3_ on airway responsiveness and inflammatory cell recruitment [11, 12, 17, 22, 29]. We used multiplex and ELISA assays to measure BAL concentrations of these inflammatory moieties in the O_3_-exposed mice. Factorial ANOVA indicated that compared to chow, HFD feeding caused a significant increase in BAL IL-33, BAL IL-5, and BAL IL-1α, and a significant *decrease* in BAL LIF, CXCL10, CXCL9, CCL2, CXCL2, and CCL4 (Fig. 8). Factorial ANOVA also indicated a significant effect of ST2 deficiency on BAL IL-33, BAL IL-5, and BAL CCL2. Significant reductions in BAL IL-5 were observed in ST2-deficient versus WT mice, whether the mice were chow- or HFD-fed (Fig. 8b). These reductions are consistent with the known ability of IL-33 to induce release of type 2 cytokines from ILC2 and other immune cells within the airways [12, 30, 31]. There was also a significant reduction in BAL CCL2 in ST2 versus WT chow-fed mice, as reported [17], but no effect of ST2 deficiency in HFD-fed mice. ST2 deficiency also increased BAL IL-33 in HFD- but not chow-fed mice (Fig. 8a). There were no significant effects of diet or genotype on BAL IL-17A, CCL11, G-CSF, IL-6, CXCL1, IL-9, or CCL3, except for an increase in CXCL1 in the ST2^−/−^ versus WT chow fed mice (Fig. S3). Other cytokines and chemokines were below the limit of detection in most mice.

### Effect of cohousing on HFD- and ST2-dependent changes in pulmonary responses to O_3_

As described above, cohousing transfers gut microbiota from one mouse to its cagemates and normalizes differences in their gut microbiota [18, 19]. For this reason, cohousing has been used as a means to evaluate effects of the gut microbiome on physiological endpoints. We have reported that in chow-fed mice, cohousing WT mice with ST2^−/−^ mice alters their gut microbiomes and abolishes ST2-dependent changes in pulmonary responses to O_3_ [17]. HFD-feeding alters the gut microbiome [28, 32] and the gut microbiome contributes to changes in the pulmonary response to O_3_ observed in female *db/db* mice [22]. Consequently, we compared the effects of ST2 deficiency on responses to O_3_ in same housed and cohoused HFD-fed mice. Because our data from antibiotic-treated and germ-free mice indicated no effect of the microbiome on the innate airway responsiveness of obesity (i.e. airway responsiveness measured in air-exposed mice [22]), effects of cohousing were explored only in O_3_-exposed mice. Note that cohousing had no effect on HFD-induced changes in body mass in either WT or ST2^−/−^ mice (Fig. S4).

### Pulmonary mechanics and airway responsiveness

**Fig. 9:**
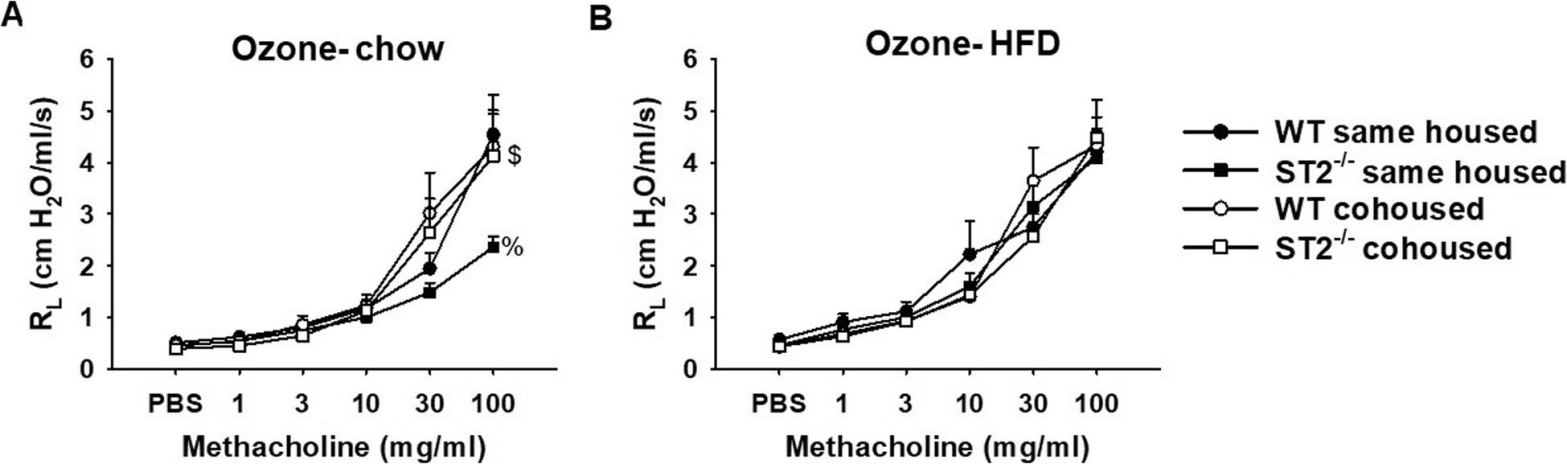
Effect of ST2 deficiency and cohousing on airway responsiveness in mice fed chow or HFD and exposed to O_3_ (2 ppm for 3 h). Results are mean + SE of 6–11 mice/group. % *p* < 0.05 versus WT mice with same diet and housing; $ versus same housed mice with same diet and genotype, as assessed using ANOVA considering either all chow-fed or all HFD-fed mice

As we have described, in chow-fed mice, cohousing abolished reductions in airway responsiveness observed in ST2^−/−^ versus WT mice exposed to O_3_ [17] (Fig. 9a). In contrast, cohousing WT and ST2^−/−^ mice had no effect on airway responsiveness in HFD-fed mice exposed to O_3_ (Fig. 9b).

### Pulmonary injury and inflammation

As we have described [17], in chow-fed mice exposed to O_3_, ST2-dependent reductions in BAL neutrophils and macrophages observed in same housed mice were no longer observed in cohoused mice (Fig. 7a, b). Cohousing had no effect on BAL neutrophils or macrophages in O_3_-exposed HFD fed mice whether the mice were WT or ST2^−/−^ (Fig. 7a, b). O_3_-induced changes in BAL cytokines and chemokines were similar in HFD-fed same housed and cohoused mice with the following exceptions (Fig. 8). Compared to same housed mice, cohousing reduced BAL IL-33 in HFD-fed ST2^−/−^ mice (Fig. 8a). In cohoused mice, BAL IL-5 was also greater in HFD-fed than chow-fed WT mice (Fig. 8b).

### Effect of ST2 deficiency and cohousing on the gut microbial community structure in chow-fed versus HFD-fed mice

**Fig. 10:**
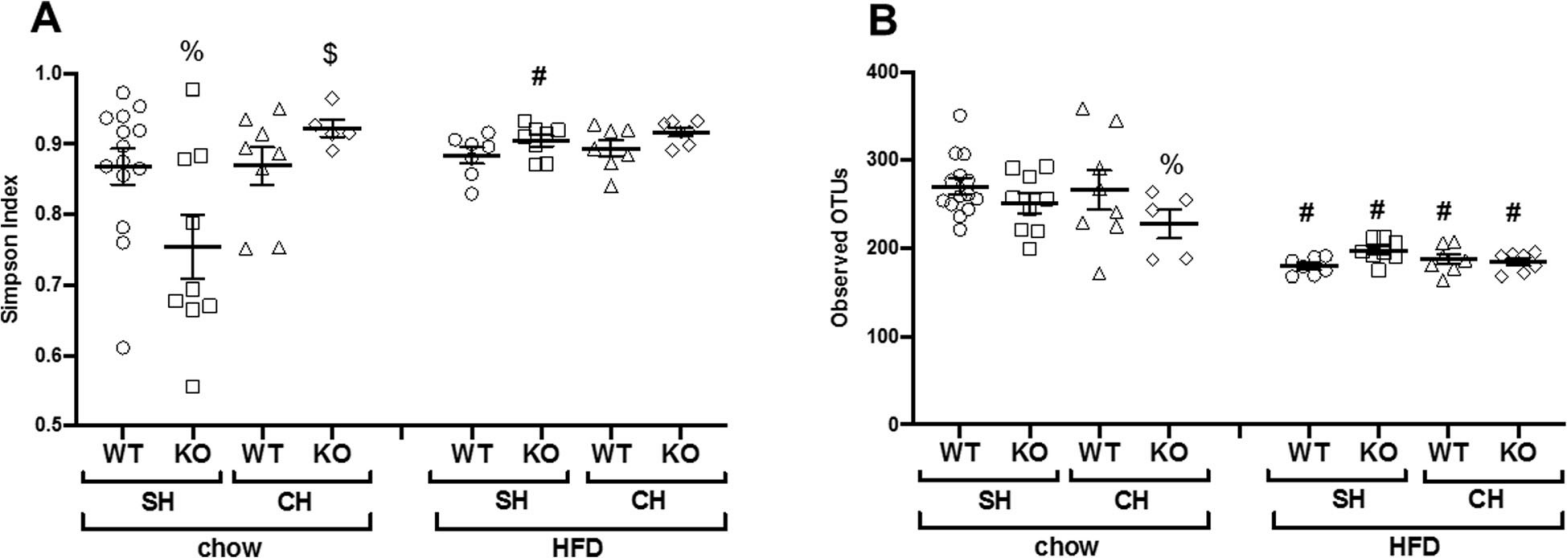
Effect of ST2 deficiency, cohousing, and diet on gut microbial richness and diversity. DNA was extracted from fecal pellets of male mice prior to exposure. Results of 16S rRNA sequencing of fecal DNA harvested from WT and ST2^−/−^ (KO) mice fed chow or HFD for 12 weeks and housed with other mice of the same or opposite (cohoused) genotype. **a** Simpson index and (**b**) Richness. In **a** and **b**, bars indicate mean ± SEM. Each symbol indicates one mouse. # *p* < 0.05 versus chow fed mice with same genotype and housing. % *p* < 0.05 versus WT mice with same diet and housing; $ *p* < 0.05 versus same housed mice with same diet and genotype. SH: same housed. CH: cohoused

We have reported that both ST2 deficiency and cohousing altered the taxonomic composition and the functional capacity of the gut microbiome in these chow-fed mice [17]. Because HFD feeding alters the gut microbiome [28, 32] and because our data indicated differences in the impact of cohousing and ST2 deficiency in chow-fed versus HFD-fed mice (Figs. 6, 7, 9), we performed 16S rRNA sequencing on fecal DNA harvested from the HFD-fed mice and compared the results to the 16S rRNA sequencing results from chow-fed mice. Fecal samples were collected before any exposure was administered. We examined the gut rather than the lung microbiome because previous data from our lab suggest that it is the gut and not the lung microbiome that accounts for the role of the microbiome in pulmonary responses to ozone [33]. In chow-fed same housed mice, the Simpson index, a measure of ecological diversity, was significantly lower in ST2-deficient than WT same housed (Fig. 10a). Cohousing reversed this effect of ST2 deficiency as described [17]. However, no such effect of ST2 deficiency was observed in HFD-fed mice (Fig. 10a). Compared to chow-fed mice, the number of operational taxonomic units (OTUs) was significantly lower in the HFD-fed mice, i.e. the number of different types of bacteria identified was lower (Fig. 10b). There was no effect of either ST2 deficiency or cohousing on this measure of richness in HFD-fed mice, but ST2 deficiency reduced richness in chow-fed mice, particularly in cohoused mice (Fig. 10b).

**Fig. 11:**
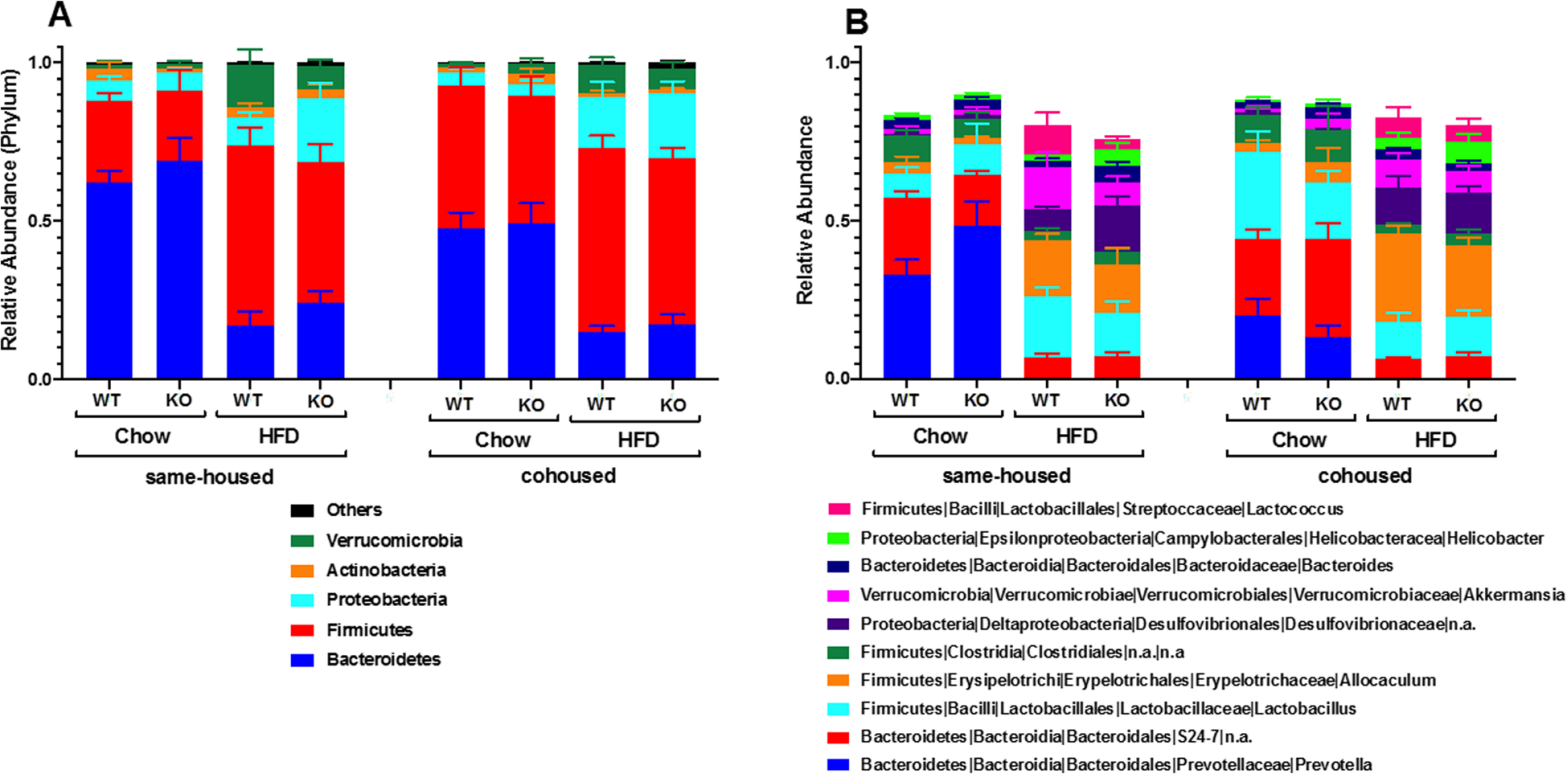
Effect of ST2 deficiency, cohousing, and diet on the gut microbial community structure. **a** Taxa relative abundances at the phylum level; **b** Top 10 genera taxa abundances relative to total gut Bacteria and Archaea reads. Error bars indicate SEM

Principal component analysis using the Bray-Curtis method indicated marked differences in the gut microbiomes of the chow- and HFD-diet fed mice whether the mice were WT or ST2^−/−^ and whether the mice were same housed or cohoused (Fig. S5). Within the chow-fed mice, some separation of the same housed versus cohoused mice was observed, as we have reported [17], but no such differences were observed in the HFD-fed mice. Examination of taxon abundance at both the phylum and genus level also indicated marked differences between the chow- and HFD-fed mice (Fig. 11). At the phylum level, these differences included a greater abundance of Firmicutes and a corresponding reduction in Bacteroidetes in the HFD- versus chow-fed mice (Fig. 11a), consistent with most reports by other investigators [20, 21]. Examination of the 10 most abundant lower order taxa indicated that these differences also included decreases in *Prevotella* and S24–7 in the HFD-fed, which accounted for much of the reduction in total Bacteroidetes, and increases in *Allobaculum, Lactococcus,* and Desulfovibrionaceae (Fig. 11b). Statistical analysis using Multivariate Association with Linear Models (MaAsLin) [26] confirmed the statistical significance of these differences and identified other less abundant taxa that were also impacted by HFD feeding (Table S1).

Within the chow-fed group of mice, MaAsLin identified a statistically significant increase in the abundance of bacteria in the *Lactobacillus* genus and a decrease in *Prevotella* in cohoused versus same housed mice (Table S2), as well as changes in a few less abundant taxa.

In contrast, within the HFD-fed group of mice, MaAsLin identified no statistically significant effect of either housing or genotype for any taxon.

### Effect of cohousing on systemic inflammation

We also examined serum cytokines and chemokines in the O_3_ exposed mice in which airway responsiveness was evaluated. Those cytokines and chemokines for which a significant effect of cohousing was observed in at least one of the groups are shown in Fig. S6. Changes in IL-5 are also provided as a positive control for the effect of ST2 deficiency. It is important to note that values obtained in these O_3_-exposed mice (Fig. S6) cannot be directly compared to the unexposed and anesthetized mice in Fig. 5 because anesthesia, methacholine challenge, and O_3_ exposure could each alter systemic inflammation. Serum IL-5 was reduced by ST2 deficiency in chow-fed mice whether the mice were same housed or cohoused (Fig. S6A). In contrast, there was no effect of ST2 deficiency on serum IL-13 (Fig. S6B). For IL-13 and for most of the other inflammatory markers for which an effect of cohousing was observed (Fig. S6B-H), the effect was an increase in the concentration of the inflammatory marker in the cohoused versus same cohoused WT chow-fed mice. These cohousing-dependent changes in systemic inflammation did not precisely parallel cohousing-dependent differences in the response to O_3_ (Figs. 9). However, given that these changes were primarily observed in the chow-fed versus HFD-fed mice (Fig. S6) and that effects of cohousing on responses to O_3_ were also observed in the chow-fed but not HFD-fed mice (Fig. 9) we cannot rule out the possibility that changes in systemic inflammation contribute to the role of microbiome in responses to O_3_ observed in the chow-fed mice.

## Discussion

Our primary goal was to determine whether IL-33 contributes to pulmonary responses to O_3_ in mice with obesity induced by HFD feeding. We have reported reductions in O_3_-induced AHR and neutrophil recruitment in lean chow-fed ST2^−/−^ versus WT mice [17]. However, in obese HFD-fed mice that were derived from the same litters, no such effect of ST2 deficiency was observed (Figs. 6b, 7a). These data indicate diet/obesity-related differences in the role of IL-33 in pulmonary responses to O_3_.

Although ST2 deficiency had no effect on O_3_-induced AHR (Fig. 6b) or neutrophil recruitment (Fig. 7a) in obese HFD-fed mice, treatment with anti-ST2 antibodies attenuates both the AHR and the neutrophil recruitment associated with O_3_-exposure in obese *db/db* mice [12]. IL-33 is known to cause release of type 2 cytokines from ILC2 and other cells within the airways [12], so the marked reductions in BAL IL-5 in ST2^−/−^ versus WT HFD-fed mice that were exposed to O_3_ (Fig. 8b) indicate that the IL-33 signaling pathway was activated in response to O_3_ even in these HFD-fed mice. It is possible that differences in the ability of ST2 deficiency to reduce responses to O_3_ in *db/db* mice [12] but not mice with DIO (Figs.6, 7) is due to differences in the sex of the mice: sex impacts responses to O_3_ [17] and the *db/db* mice were female [12] but we used male mice in this study because female mice are resistant to the induction of obesity by HFD feeding [24]. However, it is also possible that the modality of obesity contributed. For example, others have reported differences in the impact of obesity on bacterial pneumonia depending on the type of obese mice used [34]. *Db/db* are more hyperglycemic than mice with DIO and marked obesity develops much more rapidly in *db/db* than in mice with DIO [35]. *Db/db* mice are obese because they lack the longform of the receptor for leptin, a satiety hormone expressed in adipose tissue. Importantly, adipose tissue expression of both IL-33 and ST2 are observed in mice with DIO but not in *db/db* mice [36], suggesting a possible role for leptin in the regulation of the IL-33 signaling pathway. The observation that IL-33 increases the expression of the leptin receptor in some cell types [37] also suggest that there might be differences in the role of IL-33 in *db/db* mice versus mice with other forms of obesity. Such a difference could explain the impact of ST2 deficiency on responses to O_3_ in *db/db* mice but not mice with DIO.

Compared to chow-fed WT mice, HFD-fed WT mice had increases in serum concentrations of many pro-inflammatory cytokines and chemokines but reduced concentrations of IL-5 (Fig. 5). This imbalance between acute phase cytokines and type 2 cytokines has been reported by others studying adipose tissue in obesity [38]. The fact that this imbalance was also observed in serum suggests that these serum cytokines may derive from adipose tissue. Importantly, in chow-fed but not HFD-fed mice, ST2 deficiency caused significant reductions in serum IL-5. The data suggest that IL-33 maintains a type 2 dominant state in lean mice, consistent with the reports of others [38] and that these effects of IL-33 are impaired in the obese mice. However, it is unlikely that these obesity-related differences in the impact of IL-33 on systemic type 2 cytokines account for obesity-related differences in the impact of IL-33 on O_3_-induced AHR. First, in same housed mice, O_3_-induced AHR was greater in WT HFD-fed than WT chow-fed mice (Fig. 6b), even though serum IL-5 was lower in the HFD- than the chow-fed mice (Fig. 5a). Second, in the lung, ST2 deficiency reduced IL-5 in both the chow-fed and HFD-fed mice and IL-5 was actually greater in the HFD fed mice (Fig. 8b).

Instead, our data suggest a role for the microbiome in diet/obesity-related differences in the impact of IL-33 on pulmonary responses to O_3_. In chow-fed mice, cohousing the ST2^−/−^ mice with WT mice abolished differences in their response to O_3_ (Figs. 7, 9), suggesting a role for the microbiome in the effects of ST2 deficiency on pulmonary responses to O_3_ in these lean mice. The gut microbiome can affect pulmonary responses to O_3_ [33] and both ST2 deficiency and cohousing caused changes in the gut microbiome in chow-fed mice [17]. HFD-feeding caused marked changes in the gut microbiome and abolished both ST2-related and cohousing-related differences in the gut microbiome that were observed in chow-fed mice (Figs.10, 11, Fig. S5, Tables S1, S2). Moreover, whereas cohousing augmented responses to O_3_ in chow-fed ST2^−/−^ mice, no effect of cohousing was observed in HFD-fed ST2^−/−^ mice (Fig. 9). The ability of HFD feeding to override genetic impacts on the gut microbiome has been reported by others [39].

Although there was no effect of ST2 deficiency on O_3_-induced AHR in HFD-fed mice, ST2 deficiency did affect innate airway responsiveness in HFD-fed mice. Compared to air-exposed HFD-fed WT mice, airway responsiveness was significantly *increased* in air-exposed HFD-fed ST2^−/−^ mice, at least at the lowest concentrations of methacholine (Fig. 6a). In contrast, no such effect of ST2 deficiency was observed in the chow-fed mice (Fig. 6a). The data suggest that IL-33 protects against the development of innate AHR in these obese mice. Similarly, IL-33 is proposed to protect against the adipose tissue inflammation of obesity [40, 41]. Increases in IL-1β and TNFα, have been proposed to contribute to the innate AHR of obesity [42–44]. Hence, we considered the possibility that ST2 deficiency augmented innate AHR in HFD-fed mice (Fig. 6a) by augmenting these cytokines. As discussed above, we did observe increases in circulating IL-1β and TNFα, as well as increases in several other pro-inflammatory cytokines in WT HFD-fed versus chow-fed mice, but ST2 deficiency ablated rather augmented HFD-related increases in serum IL-1β and TNFα (Fig. 5a, b).

There were both strengths and weaknesses to this study. An important strength was the breeding and housing strategy. Both the chow-fed and the HFD-fed mice derived from the same litters. Most of the WT and ST2^−/−^ were also derived from the same litters because we predominantly bred ST2^+/−^ mice. Thus, mice were inoculated with the same microbiome at birth and the environmental conditions extant in the cages of the various groups of mice from birth to weaning were matched. Our data emphasize the need for attention to mouse housing conditions and the microbiome in any study of the impact of genetic deficiencies in the setting of HFD feeding.

Regarding weaknesses, it is important to note that we examined only the gut microbiome. We cannot rule out the possibility that changes in either the lung or oral microbiome also impacted responses to O_3_. We examined the gut microbiome because we have previously reported that it is the gut rather than the lung microbiome that accounts for effects of antibiotics and germ free conditions on responses to O_3_ [33]: in lean male mice, oral vancomycin reduces O_3_-induced AHR [33] but does not affect the lung microbiome [45], even though it does affect the gut microbiome [33].

Another potential weakness relates to the time point after initiation of HFD feeding at which the mice were examined. Although we observed greater effects of O_3_ on airway responsiveness, and on BAL IL-5 and IL-1α in HFD- versus chow-fed mice (Figs.6b, 8b, c), there was no effect of HFD feeding on BAL neutrophils (Fig. 7a) and most other BAL cytokines and chemokines were actually lower in HFD- versus chow-fed mice exposed to O_3_ (Fig. 8). In contrast, we have previously reported that compared to mice fed low fat diets, O_3_-induced increases in BAL concentrations of most acute phase cytokines and chemokines as well as total BAL neutrophils are greater in mice fed HFD for 30–35 weeks [16]. The difference is likely related to the time point after the onset of HFD feeding at which the mice were evaluated (12 weeks in this study), since mice evaluated 20 weeks after the onset of HFD feeding also failed to demonstrate obesity-related increases in O_3_-induced neutrophil recruitment and cytokine release [16]. Likely the duration as well as the extent of obesity is important in determining the response to O_3_. Hence it is possible that the impact of ST2 deficiency could be different in mice with DIO of more prolonged duration.

## Conclusions

Our data indicate that IL-33 contributes to O_3_-induced AHR and cellular inflammation in lean chow-fed but not obese HFD-fed male mice. Our data also support a role for the gut microbiome in obesity/diet-related differences in the impact of IL-33 on pulmonary responses to O_3_. Overweight and obese individuals now make up the majority of the US population. Our results highlight obesity-related differences in the regulation of responses to O_3_ by IL-33, and emphasize the need for attention to the role of obesity and the gut microbiome in the development of asthma pharmaceuticals, including but potentially not limited to those that target the IL-33/ST2 pathway.

## Supplementary information

**Additional file 1: Figure S1.** Effect of HFD, ST2 deficiency, and cohousing on baseline pulmonary resistance (R_L_). Shown are baseline pulmonary resistance (R_L_), Newtonian resistance (Rn), and the coefficients of lung tissue damping (G) and elastance (H) in WT and ST2^−/−^ mice fed chow or HFD and exposed to air or O_3_ (2 ppm for 3 h). For the O_3_-exposed mice, the WT and ST2^−/−^ mice were housed either with other mice of the same genotype (same housed) or with mice of the opposite genotype (cohoused). Results are mean + SE of 6–11 mice/group. * *p* < 0.05 versus air exposed mice of the same genotype, diet, and housing; # *p* < 0.05 versus chow fed mice with same genotype, housing, and exposure; % *p* < 0.05 versus WT mice with same exposure, diet, and housing; $ versus same housed mice with same exposure, diet, and genotype. Note that only same housed mice were studied with air exposure. **Figure S2.** Effect of HFD and ST2 deficiency on O_3_-induced airway hyperresponsiveness. Shown are changes in Newtonian resistance (Rn) (panels A, D), and the coefficients of lung tissue damping (G) (panels B,E) and elastance (H) (panels C,F) induced by inhaled aerosolized methacholine in WT and ST2^−/−^ mice exposed to air (panels A-C) or O_3_ (panels D-F) and examined 24 h after exposure. All mice were housed with other mice of the same genotype, as outlined in Fig. 1. Results are mean + SE of 6–11 mice/group. * *p* < 0.05 versus air exposed mice of same genotype and diet; # *p* < 0.05 versus chow fed mice with same genotype and exposure; % *p* < 0.05 versus WT mice with same exposure and diet. **Figure S3.** Effect of HFD, ST2 deficiency, and cohousing on BAL inflammatory mediators in O_3_ exposed mice. Shown are BAL concentrations of A) IL-17A, B) CCL11, C) G-CSF, D) IL-6, E) CXCL1, F) IL-9, and G) CCL3 in WT and ST2^−/−^ mice fed chow or HFD for 12 weeks from weaning and then exposed to O_3._ Mice were housed with other mice of the same or opposite (cohoused) genotype. Results are the mean + SE of 4–7 mice/group. % *p* < 0.05 versus WT mice with same diet and housing. **Figure S4.** Effect of cohousing on HFD induced increases in body mass. At weaning, wildtype (WT) and ST2 deficient (ST2^−/−^) mice were placed either on high fat diets (HFD) in which 60% of calories derived from fat in the form of lard, or on regular chow. Mice were housed with other mice of the same genotype (same housed) or with mice of the opposite genotype (cohoused). Results are mean + SE of 6–19 mice/group. # Factorial ANOVA indicated a significant (*p* < 0.05) effect of diet. **Figure S5.** Principal coordinate analysis of the gut microbiome. PCoA was calculated using the Bray-Curtis method on fecal DNA from 5 to 14 mice per group. **Figure S6.** Serum cytokines and chemokines in ozone exposed WT and ST2^−/−^ mice fed chow or HFD for 12–13 weeks. Shown are serum concentrations of A) IL-5, B) IL-13, C) CXCL1, D) G-CSF, E) IFNγ, F) TNFα, G) CXCL10, and H) CCL4 in same housed and cohoused mice. Results are the mean + SE of 5–10 mice/group. # *p* < 0.05 versus chow-fed mice with same genotype and housing; % *p* < 0.05 versus WT mice with the same diet and housing; $ *p* < 0.05 versus same housed mice with same genotype and diet.

**Additional file 2: Table S1.** Bacterial taxa that were significantly different in chow- versus HFD-fed mice. Weanling mice were fed standard mouse chow or high fat diets for 12 weeks after which a fecal pellet was collected. Fecal DNA was extracted from the pellets and 16S rRNA sequencing was performed. Table indicates outcome from a comparison of all chow-fed versus all HFD-fed mice regardless of whether they were WT or ST2^−/−^ or whether they were same housed or cohoused. Only taxa with *p* < 0.05 and q < 0.25 are shown. A negative coefficient indicates that HFD feeding reduced the abundance of the taxon and a positive value indicates that HFD feeding increased the abundance of the taxon. Fecal samples from 64 mice (35 chow and 29 HFD) were analyzed. N not zero indicates the number of samples in which the taxon of interest was detected.

**Additional file 3: Table S2.** Bacterial taxa that were significantly affected by housing in chow-fed mice. Table indicates outcome from a comparison within the chow fed mice of all same housed versus cohoused mice. Only taxa with *p* < 0.05 and q < 0.25 are shown. A negative coefficient indicates that compared to cohoused mice, same housed mice had reduced abundance of the taxon and a positive value indicates that same housed mice had a greater abundance of the taxon. Fecal samples from 35 mice chow fed mice were analyzed. N not zero indicates the number of samples in which the taxon of interest was detected.

## Data Availability

Sequencing raw data (Fastaq) and metadata files have been deposited at the National Institute of Health – Sequence Read Archive (SRA) with accession number PRJNA516522 (biosample_accession SAMN10790706 – SAMN10790770). Other datasets used and analyzed in this study are available from the corresponding author on reasonable request.

## Abbreviations

AHR: Airway hyperresponsiveness
ANOVA: Analysis of variance
BAL: Bronchoalveolar lavage
CCL2: Chemokine (C-C motif) ligand
CXCL: Chemokine (C-X-C motif) ligand
DIO: Diet induced obesity
ELISA: Enzyme linked immunosorbent assay
G-CSF: Granulocyte-colony stimulating factor
GIP: Gastrin inhibitory peptide
HFD: High fat diet
IL: Interleukin
ILC: Innate lymphoid cell
LIF: Leukemia inhibitor factor
MaAsLin: Multivariate Association with Linear Model
O_3_: Ozone
OTU: Operational Taxonomic Unit
PYY: Peptide YY
R_L_: Pulmonary resistance
rRNA: ribosomal RNA
ST2: Interleukin 1 receptor-like 1
TNF: Tumor necrosis factor
WT: Wildtype
