# Supplementary material for: IL-33, diet-induced obesity, and pulmonary responses to ozone
Source: Respir Res. 2020 Apr 23;21:98. doi: 10.1186/s12931-020-01361-9 (PMC7181525; doi:10.1186/s12931-020-01361-9)
Supplement: Supplementary file 1 — Additional file 1: Figure S1. Effect of HFD, ST2 deficiency, and cohousing on baseline pulmonary resistance (RL). Shown are baseline pulmonary resistance (RL), Newtonian resistance (Rn), and the coefficients of lung tissue damping (G) and elastance (H) in WT and ST2−/− mice fed chow or HFD and exposed to air or O3 (2 ppm for 3 h). For the O3-exposed mice, the WT and ST2−/− mice were housed either with other mice of the same genotype (same housed) or with mice of the opposite genotype (cohoused). Results are mean + SE of 6–11 mice/group. * p < 0.05 versus air exposed mice of the same genotype, diet, and housing; # p < 0.05 versus chow fed mice with same genotype, housing, and exposure; % p < 0.05 versus WT mice with same exposure, diet, and housing; $ versus same housed mice with same exposure, diet, and genotype. Note that only same housed mice were studied with air exposure. Figure S2. Effect of HFD and ST2 deficiency on O3-induced airway hyperresponsiveness. Shown are changes in Newtonian resistance (Rn) (panels A, D), and the coefficients of lung tissue damping (G) (panels B,E) and elastance (H) (panels C,F) induced by inhaled aerosolized methacholine in WT and ST2−/− mice exposed to air (panels A-C) or O3 (panels D-F) and examined 24 h after exposure. All mice were housed with other mice of the same genotype, as outlined in Fig. 1. Results are mean + SE of 6–11 mice/group. * p < 0.05 versus air exposed mice of same genotype and diet; # p < 0.05 versus chow fed mice with same genotype and exposure; % p < 0.05 versus WT mice with same exposure and diet. Figure S3. Effect of HFD, ST2 deficiency, and cohousing on BAL inflammatory mediators in O3 exposed mice. Shown are BAL concentrations of A) IL-17A, B) CCL11, C) G-CSF, D) IL-6, E) CXCL1, F) IL-9, and G) CCL3 in WT and ST2−/− mice fed chow or HFD for 12 weeks from weaning and then exposed to O3. Mice were housed with other mice of the same or opposite (cohoused) genotype. Results are the mean + SE of 4–7 mice/group [file 12931_2020_1361_MOESM1_ESM.docx]

Online supplement for:

IL-33, diet-induced obesity, and pulmonary responses to ozone

David I. Kasahara and Stephanie A. Shore

Department of Environmental Health, Harvard T.H. Chan School of Public Health, Boston, MA 02115-6021

**Table of Contents:**

**Figure S1:**

**Figure S2**

**Figure S3**

**Figure S4**

**Figure S5**

**Figure S6**

**Table S1**

**Table S2**

**
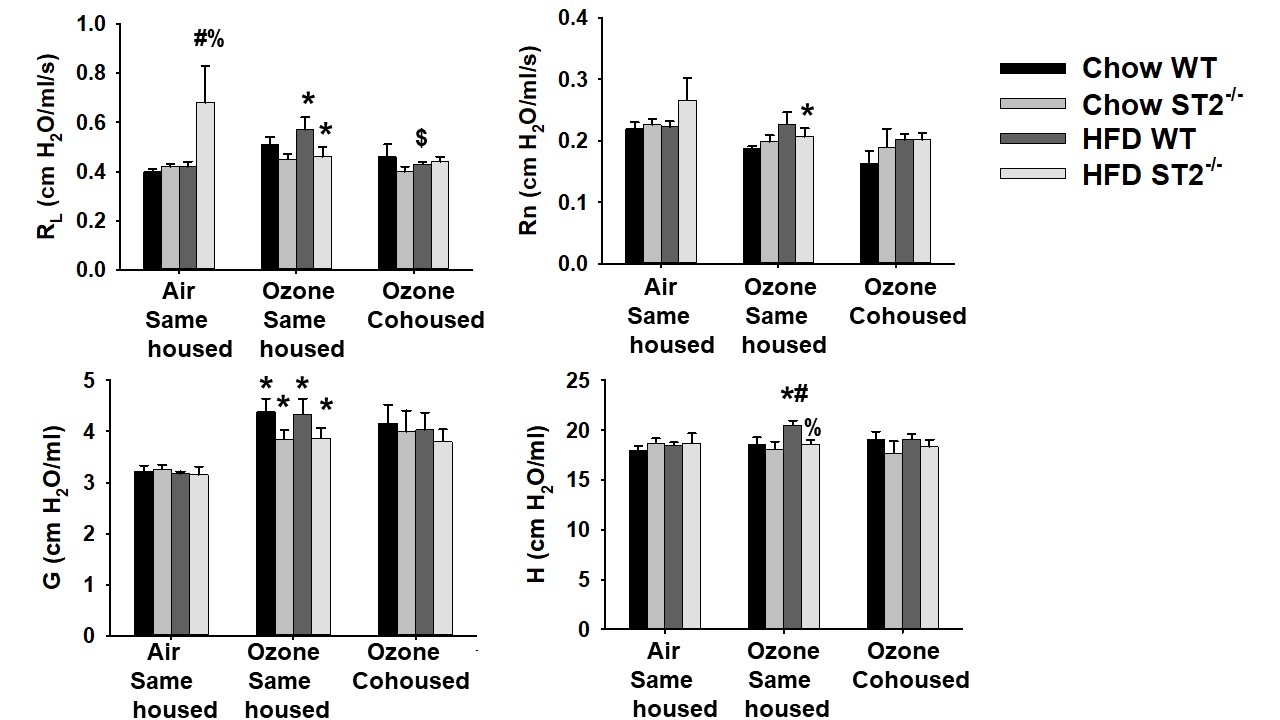
**

**Figure S1: Effect of HFD, ST2 deficiency, and cohousing on baseline pulmonary resistance (R_L_).** Shown are baseline pulmonary resistance (R_L_), Newtonian resistance (Rn), and the coefficients of lung tissue damping (G) and elastance (H) in WT and ST2^-/-^ mice fed chow or HFD and exposed to air or O_3_ (2 ppm for 3 h). For the O_3_-exposed mice, the WT and ST2^-/-^ mice were housed either with other mice of the same genotype (same housed) or with mice of the opposite genotype (cohoused). Results are mean + SE of 6-11 mice/group. * p<0.05 versus air exposed mice of the same genotype, diet, and housing; # p<0.05 versus chow fed mice with same genotype, housing, and exposure; % p<0.05 versus WT mice with same exposure, diet, and housing; $ versus same housed mice with same exposure, diet, and genotype. Note that only same housed mice were studied with air exposure.


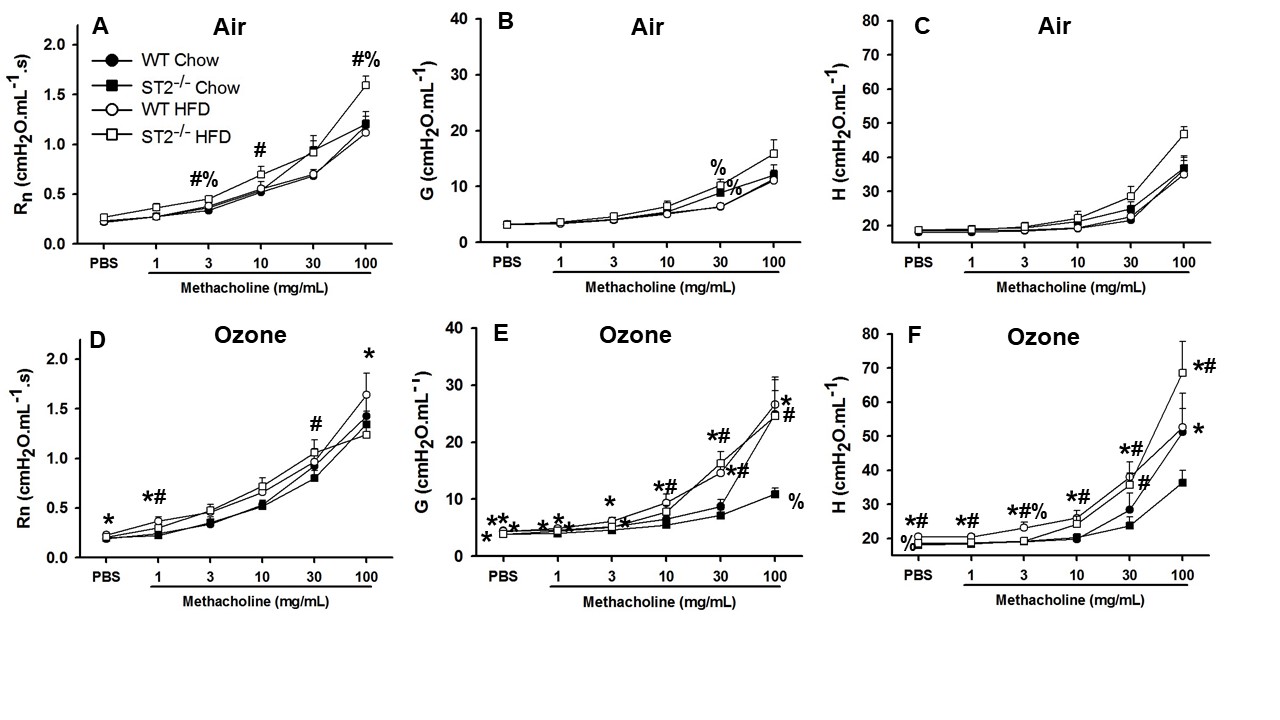


**Figure S2:** **Effect of HFD and ST2 deficiency on O_3_-induced airway hyperresponsiveness.** Shown are changes in Newtonian resistance (Rn) (panels A,D), and the coefficients of lung tissue damping (G)(panels B,E) and elastance (H)(panels C,F) induced by inhaled aerosolized methacholine in WT and ST2^-/-^ mice exposed to air (panels A-C) or O_3_ (panels D-F) and examined 24 hours after exposure. All mice were housed with other mice of the same genotype, as outlined in Figure 1. Results are mean + SE of 6-11 mice/group. * p<0.05 versus air exposed mice of same genotype and diet; # p<0.05 versus chow fed mice with same genotype and exposure; % p<0.05 versus WT mice with same exposure and diet.

**
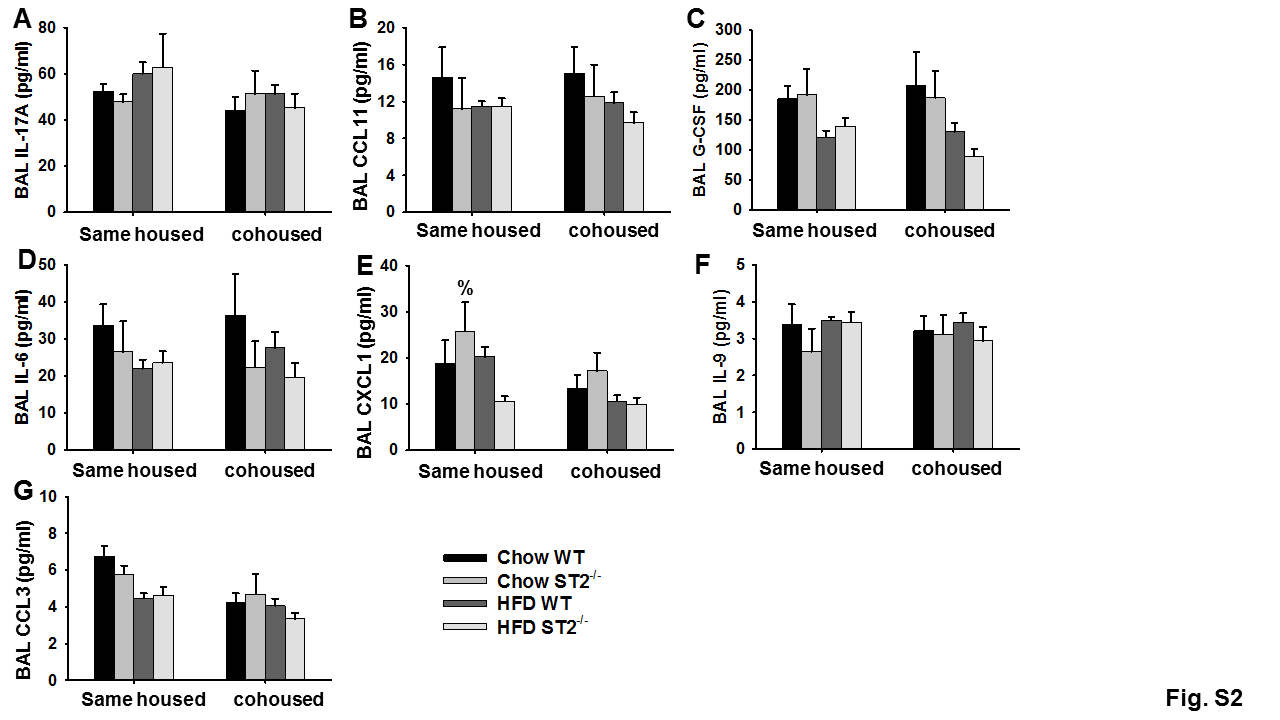
**

**Figure S3:** **Effect of HFD, ST2 deficiency, and cohousing on BAL inflammatory mediators in O_3_ exposed mice.** Shown are BAL concentrations of A) IL-17A, B) CCL11, C) G-CSF, D) IL-6, E) CXCL1, F) IL-9, and G) CCL3 in WT and ST2^-/-^ mice fed chow or HFD for 12 weeks from weaning and then exposed to O_3._  Mice were housed with other mice of the same or opposite (cohoused) genotype. Results are the mean + SE of 4-7 mice/group. % p<0.05 versus WT mice with same diet and housing.

**
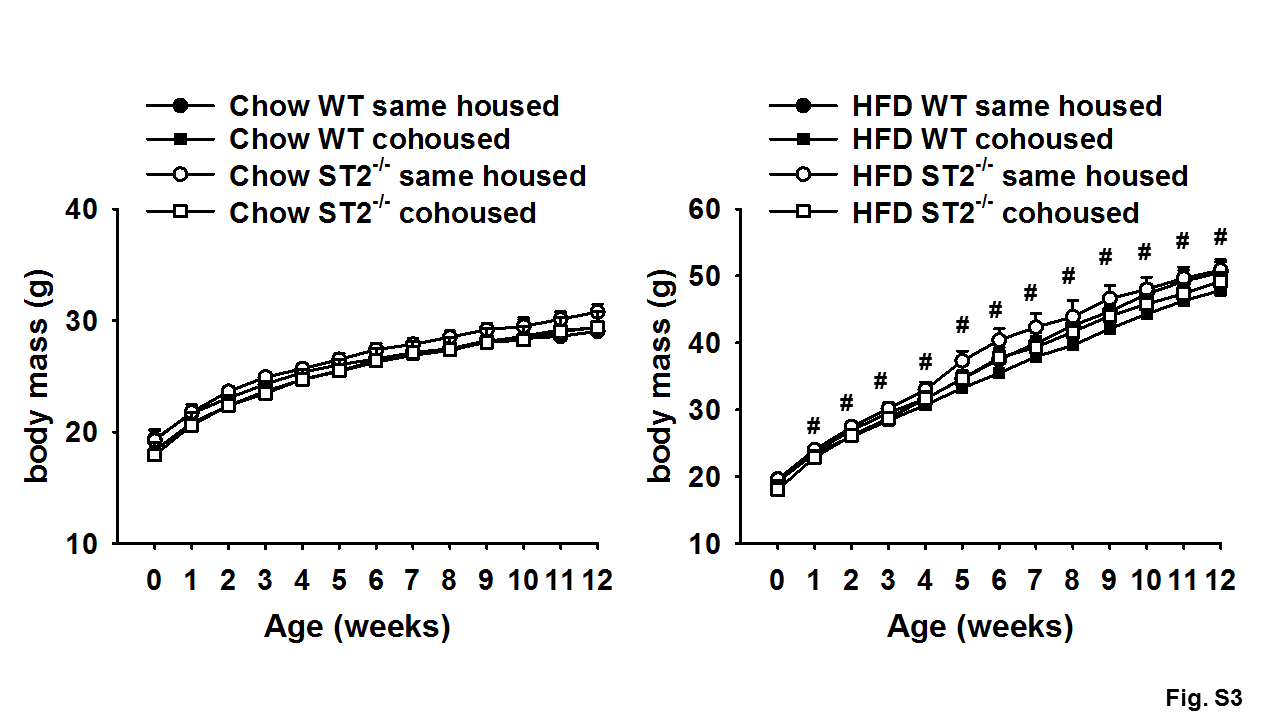
Figure S4: Effect of cohousing on HFD induced increases in body mass.** At weaning, wildtype (WT) and ST2 deficient (ST2^-/-^) mice were placed either on high fat diets (HFD) in which 60% of calories derived from fat in the form of lard, or on regular chow. Mice were housed with other mice of the same genotype (same housed) or with mice of the opposite genotype (cohoused). Results are mean + SE of 6-19 mice/group. # Factorial ANOVA indicated a significant (p<0.05) effect of diet


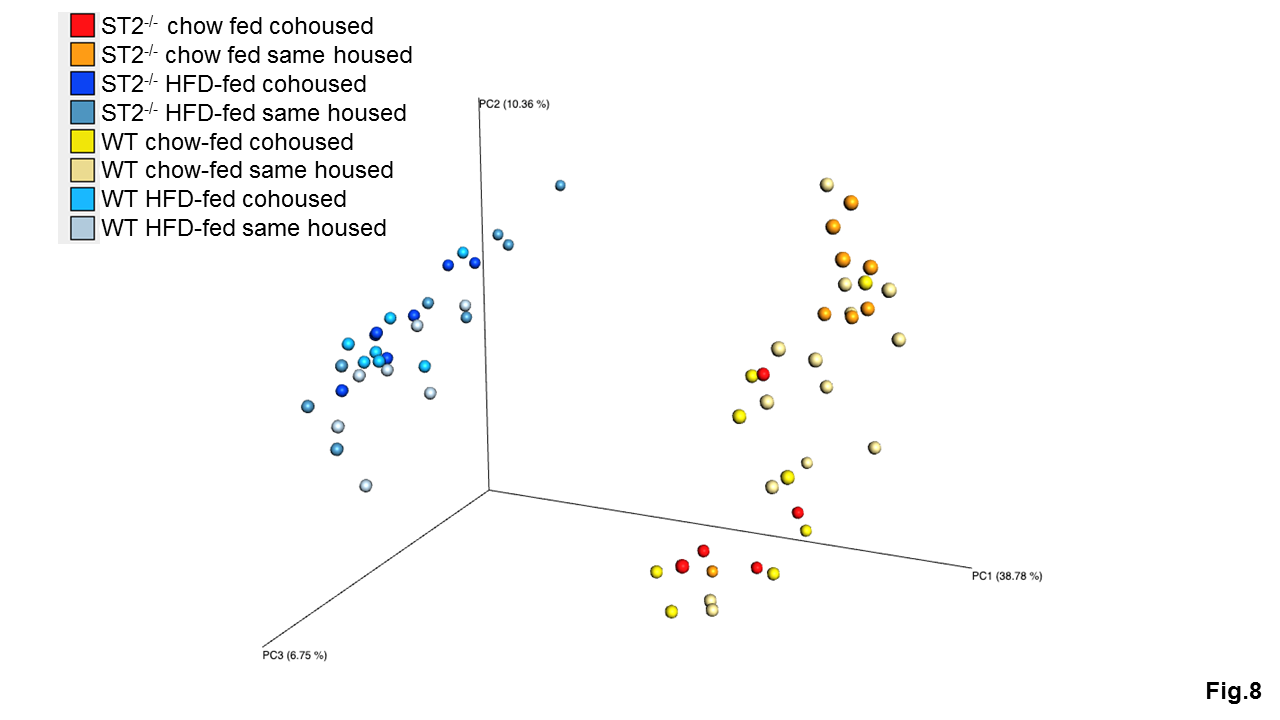


**Figure S5: Principal coordinate analysis of the gut microbiome.** PCoA was calculated using the Bray-Curtis method on fecal DNA from 5-14 mice per group.


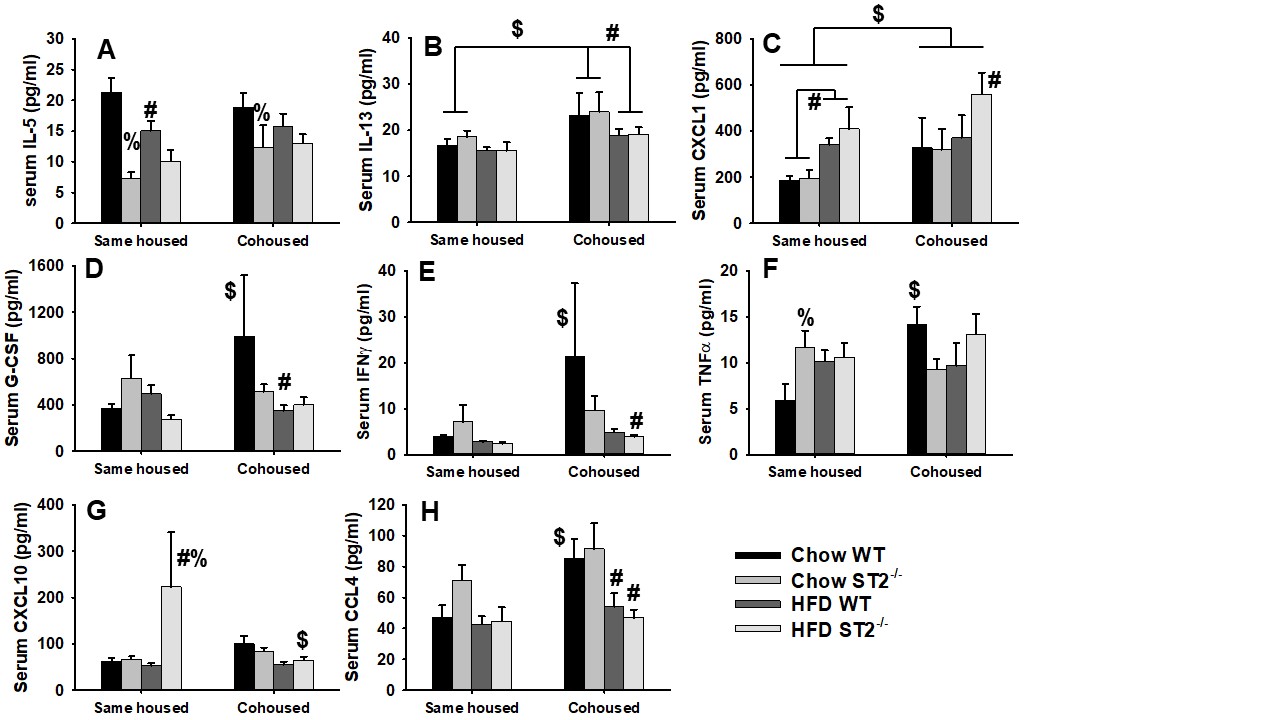


**Figure S6. Serum cytokines and chemokines in ozone exposed WT and ST2^-/-^ mice fed chow or HFD for 12-13 weeks**. Shown are serum concentrations of A) IL-5, B) IL-13, C) CXCL1, D) G-CSF, E) IFNγ, F) TNFα, G) CXCL10, and H) CCL4 in same housed and cohoused mice. Results are the mean + SE of 5-10 mice/group. # p<0.05 versus chow-fed mice with same genotype and housing; % p<0.05 versus WT mice with the same diet and housing; $ p<0.05 versus same housed mice with same genotype and diet.
